# General Nonlocal Probability of Arbitrary Order

**DOI:** 10.3390/e25060919

**Published:** 2023-06-10

**Authors:** Vasily E. Tarasov

**Affiliations:** 1 Skobeltsyn Institute of Nuclear Physics, Lomonosov Moscow State University, Moscow 119991, Russia; tarasov@theory.sinp.msu.ru; 2 Department of Physics, 915, Moscow Aviation Institute (National Research University), Moscow 125993, Russia

**Keywords:** general fractional calculus, fractional derivatives, fractional integrals, nonlocal probability, probability theory, 60Axx, 26A33, 60A99, 60E05

## Abstract

Using the Luchko’s general fractional calculus (GFC) and its extension in the form of the multi-kernel general fractional calculus of arbitrary order (GFC of AO), a nonlocal generalization of probability is suggested. The nonlocal and general fractional (CF) extensions of probability density functions (PDFs), cumulative distribution functions (CDFs) and probability are defined and its properties are described. Examples of general nonlocal probability distributions of AO are considered. An application of the multi-kernel GFC allows us to consider a wider class of operator kernels and a wider class of nonlocality in the probability theory.

## 1. Introduction

Mathematical theory of integral and differential operators of AOs is well developed [1,2,3,4,5,6,7]. This theory has a long history [1,8,9,10,11,12,13]. Fractional calculus is widely used to describe various phenomena and processes with non-locality in time and space (for example, see books [14,15,16,17,18,19,20,21,22,23] and handbooks [24,25]). Fractional operators of arbitrary (integer and non-integer) orders have various probabilistic interpretations that are proposed in [26,27,28,29,30,31]. It should be noted that the relationship between fractional calculus and probability theory (PT) is also well-known (for example, see [32,33,34,35,36]).

In fractional calculus, the power-law types of operator kernels are usually used. To take into account wider forms of non-localities, general fractional calculus (GFC) has been proposed. This calculus is based on the Sonin’s results [37,38] and its extensions starting from 1884. A new stage of development of GFC began with the Kochubei’s work in 2011 [39,40,41], in which this term was proposed. The next most important stage in development of GFC began with the Luchko’s work in 2021 [42]. In the past two years, the Luchko’s GFC has been actively developed in works [42,43,44,45,46,47,48,49,50,51,52,53]. Among the works devoted to the development of GFC and its application in mathematics, the following works should be also noted [54,55,56,57,58,59,60,61,62,63,64,65,66,67]. Applications of GFC in physics and other sciences are considered in [68,69,70,71,72,73,74,75,76,77,78,79,80,81]. Trends in the development and applications of GFC and some open problems of GFC are described in Sections 2 and 4 of review [82]. It should be noted that an application of the GFC to extend the probability theory for a nonlocal case and then apply this theory to nonlocal statistical physics was first proposed in works [65,80].

The mathematical motivation for this article is an extension of nonlocal and general fractional probability theory (PT) to nonlocal multi-kernel case of AO. This extension can be considered as a generalization of the fractional PT of AO with power-law kernels [81] and the nonlocal PT [65] that is based on the single-kernel GFC of first order. The physical motivation of such a generalization of PT is the development of nonlocal statistical mechanics and the expansion of types of nonlocalities, which can be accountable in the description of statistical physical systems.

Let us describe in more detail the mathematical motivation of the extension of nonlocal PT to nonlocal case of AO. For the first time, a generalization of the standard PT (SPT) for nonlocal case was proposed in the work [65] in 2022, where the Luchko GFC of the first order was applied. Recently, a generalization of the SPT for a nonlocal case for arbitrary order was proposed in work [81]. However, in this paper, nonlocality is considered only in the power-law form and the GFC is not used. By virtue of this, the need to expand the nonlocal PT to an arbitrary order arises. For such an extension, it is proposed to use the multi-kernel GFC of AO, which is suggested in [53]. It can also be said that one of the mathematical motivations is a generalization of the SPT similar to the GFC, which is a generalization of the standard calculus of integrals and derivatives of arbitrary integer orders.

In Section 2, (preliminaries), a multi-kernel GFC of AO is described. In Section 3, nonlocal and GF PDFs, nonlocal and GF CDFs with its properties are considered. Nonlocal and GF probability of AOs on intervals (0,∞) and (a,b) with −∞<a<b<∞ are described. In Section 4, example of nonlocal distribution of AO on interval [0,∞) is proposed. In Section 5, examples of nonlocal distributions on finite interval [a,b] is described. A brief conclusion is given in Section 6.

## 2. Preliminaries: GFC of AO

In this section, some elements of single-kernel GFC [42,43,45,46,47] and multi-kernel GFC [53] are described.

### 2.1. Single-Kernel GFC of AO

Let us define the function spaces $C_{-1}\left(0,\infty \right)$ and $C_{-1,0}\left(0,\infty \right)$ [42,83].

**Definition** **1.** (Set $C_{-1}\left(0,\infty \right)$). *Let a function f(x) be represented in the form*

(1) $$f\left(x\right)\;=\;x^{p}\;g\left(x\right),\;\;\left(p\;>\;-1\right)$$

*for all x>0, where g(x)∈C[0,∞).*

*The set of such functions is denoted as $C_{-1}\left(0,\infty \right)$.*

**Definition** **2.** (Set $C_{-1,0}\left(0,\infty \right)$). *Let a function K(x) be represented in the form*

(2) $$K\left(x\right)\;=\;x^{q}\;k\left(x\right),\;\left(-1\;<\;q\;<\;0\right)$$

*for all x>0, where k(x)∈C[0,∞).*

*The set of such functions is denoted as $C_{-1,0}\left(0,\infty \right)$.*

Let us define a set of the kernels that satisfy condition (3) and belong to the special spaces of functions.

**Definition** **3.** (Luchko set of operator kernel pairs). *Let functions M(x) and K(x) satisfy the following conditions.*

*(1) M(x) and K(x) satisfy the Luchko condition kernels M(x) and K(x) should be satisfied [43] in the form*

(3) $$\left(M\;*\;K\right)\left(x\right)\;=\;{\left\{1\right\}}^{n}\left(x\right)\;=\;h_{n}\left(x\right)\;\mathrm{for}\;\mathrm{all}\;\;\;x\;>\;0,$$

*where n∈N and*

$$h_{\alpha }\left(x\right)\;=\;\frac{x^{\alpha -1}}{\Gamma (\alpha )}\;\left(\alpha \;>\;0\right)$$

*where $h_{1}\left(x\right)=\left\{1\right\}\left(x\right)$ is the Heaviside step function.*

*(2) The kernel M(x) of GFI belongs to the space $C_{-1}\left(0,\infty \right)$.*

*(3) The kernel K(x) of GFD belongs to the space $C_{-1,0}\left(0,\infty \right)$.*

*The set of such kernel pairs (M,K) is called the Luchko set and is denoted by $L_{n}$.*

**Theorem** **1.** *Let (M,K) be a pair of the Luchko set $L_{1}$.*

*Then, the pair $\left(M_{n},\;K_{n}\right)$ of the kernels, which are given by the equations*

(4) $$M_{n}\left(x\right)\;=\;\left({\left\{1\right\}}^{n-1}\;*\;M\right)\left(x\right),\;K_{n}\left(x\right)\;=\;K\left(x\right),$$

*belongs to the Luchko set $L_{n}$.*

**Proof.**  Theorem 1 is proved in [43]. □

Let us define GF integrals (GFIs) and GF derivatives (GFDs) of AOs in the framework of the Luchko GFC that is proposed in paper [43].

**Definition** **4** (GF operators of AOs)**.** *Let (M,K) be a pair of the kernels from the Luchko set $L_{n}$.*

*The GFI with the kernel M(x) for the function $f\left(x\right)\in C_{-1}\left(0,\infty \right)$ is defined by the equation*

(5) $$I_{\left(M\right)}^{x}\left[u\right]\;f\left(u\right)\;:=\;\int_{0}^{x}M\left(x-u\right)\;f\left(u\right)\;du,$$

*where x>0.*

*The Riemann–Liouville type of GFD of AO with the kernel K(x) for the function $f\left(x\right)\in C_{-1}\left(0,\infty \right)$ is defined as*

(6) $$D_{\left(K\right)}^{x}\left[u\right]\;f\left(u\right)\;:=\;\frac{d^{n}}{dx^{n}}\;\int_{0}^{x}K\left(x-u\right)\;f\left(u\right)\;du,$$

*where n∈N, and x>0.*

*The Caputo type of GFD of AO with the kernel K(x) for the function $f^{\left(n\right)}\left(x\right)\in C_{-1}\left(0,\infty \right)$ is defined as*

(7) $$D_{\left(K\right)}^{x,*}\left[u\right]\;f\left(u\right)\;:=\;I_{\left(K\right)}^{x}\left[u\right]\;f^{\left(n\right)}\left(u\right)\;=\;\int_{0}^{x}K\left(x-u\right)\;f^{\left(n\right)}\left(u\right)\;du,$$

*where n∈N, x>0 and $f^{\left(n\right)}\left(u\right)=d^{n}f\left(u\right)/du^{n}$.*

**Definition** **5.** *Let a function K(x) belong to the space $C_{-1}\left(0,\infty \right)$, and let a function f(x) can be represented in the form*

(8) $$f\left(x\right)\;=\;I_{\left(K\right)}^{x}\left[u\right]\;\varphi \left(u\right),$$

*for all x>0, where $\varphi \left(x\right)\;\in \;C_{-1}\left(0,\infty \right)$.*

*Then, the set of such functions f(x) is denoted as $C_{-1,(K)}\left(0,\infty \right)$.*

Let us give some important properties of the GFIs of AO.

**Theorem** **2** (Properties of GF integrals)**.** *Let (M,K) be a pair of the kernels from the Luchko set $L_{n}$, and let a function f(x) belong to the set $C_{-1}\left(0,\infty \right)$.*

*Then the GFI of this function is also belongs to the set, i.e., $I_{\left(M\right)}^{x}\left[u\right]\;f\left(u\right)\;\in \;C_{-1}\left(0,\infty \right)$, and*

(9) $$I_{\left(M\right)}^{x}:\;C_{-1}\left(0,\infty \right)\;\to \;C_{-1}\left(0,\infty \right),$$

*and the semi-group property is satisfy*

(10) $$I_{\left(M_{1}\right)}^{x}\left[u\right]\;I_{\left(M_{2}\right)}^{u}\left[w\right]\;f\left(w\right)\;=\;I_{\left(M_{1}*M_{2}\right)}^{x}\left[u\right]\;f\left(u\right)$$

*for all x>0.*

The fundamental theorems (FT) of Luchko’s GFC, which are proved in [43], describes basic properties of GFIs and GFDs of AOs.

**Theorem** **3** (First FT for GFD of AO)**.** *Let (M,K) be a pair of the kernels from the Luchko set $L_{n}$.*

*Then, for the GFD of the Riemann–Liouville type, the equation*

(11) $$D_{\left(K\right)}^{x}\left[u\right]\;I_{\left(M\right)}^{u}\left[w\right]\;f\left(w\right)\;=\;f\left(x\right)$$

*holds for all x>0, if the function f(x) belongs to the space $C_{-1}\left(0,\infty \right)$.*

*Then, for the GFD of the Caputo type, the equation*

(12) $$D_{\left(K\right)}^{x,*}\left[u\right]\;I_{\left(M\right)}^{u}\left[w\right]\;f\left(w\right)\;=\;f\left(x\right)$$

*holds for all x>0, if the function f(x) belongs to the space $C_{-1,(K)}\left(0,\infty \right)$.*

**Theorem** **4** (Second FT Theorem GFD of AO)**.** *Let (M,K) be a pair of the kernels from the Luchko set $L_{n}$.*

*Then, for the GFD of Riemann–Liouville type, the equation*

(13) $$I_{\left(M\right)}^{x}\left[u\right]\;D_{\left(K\right)}^{u}\left[w\right]\;F\left(u\right)\;=\;F\left(x\right)$$

*holds for all x>0, if function F(x) belong to the set $C_{-1,(M)}\left(0,\infty \right)$.*

*Then, for the GFD of the Caputo type, the equation*

(14) $$I_{\left(M\right)}^{x}\left[u\right]\;D_{\left(K\right)}^{u,*}\left[w\right]\;F\left(w\right)\;=\;F\left(x\right)\;-\;\sum_{k=0}^{n-1}F^{\left(k\right)}\left(0\right)\;h_{k+1}\left(x\right)$$

*holds for all x>0, if function F(x) belong to the space $C_{-1}^{n}\left(0,\infty \right)$, i.e., $F^{\left(n\right)}\left(x\right)\in C_{-1}\left(0,\infty \right)$, where $F^{\left(k\right)}\left(x\right)=d^{k}F\left(x\right)/dx^{k}$.*

### 2.2. Multi-Kernel GFC of AO

The multi-kernel GFC is proposed in [53] to expand the GFC to the simultaneous use of different operator kernels.

For the case $M_{j}\left(x\right)\;=\;M\left(x\right)$ for all j=1,…,m, the multi-kernel GFC gives the single-kernel GFC proposed by Luchko [43,45,46,47].

Let us define the convolutional product $M^{<1|m>}\left(x\right)$ and $K^{<1|m>}\left(x\right)$ with m∈N by the equation

(15) $$M^{<1|m>}\left(x\right)\;=\;\left(M_{1}\;*\;\ldots \;*\;M_{m}\right)\left(x\right),$$

(16) $$K^{<m|1>}\left(x\right)\;=\;\left(K_{m}\;*\;\ldots \;*\;K_{1}\right)\left(x\right),$$

where $K_{j}\left(x\right),\;M_{j}\left(x\right)\;\in \;C_{-1}\left(0,\infty \right)$ for all j=1,…,m. If $M_{j}\left(x\right)\;=\;M\left(x\right)$ for all j=1,…,m, them the convolutional product $M^{<1|m>}\left(x\right)$ is the convolutional power $M^{<m>}\left(x\right)$.

**Theorem** **5** (Commutative ring $\left(C_{-1}\left(0,\infty \right),+,*\right)$)**.** *The triple $R_{-1}=\left(C_{-1}\left(0,\infty \right),+,*\right)$ with the standard addition + and multiplication ∗ in form of the Laplace convolution is a commutative ring without divisors of zero.*

**Proof.**  Theorem 5 is proved in [83]. □

Using Theorem 5, one can state that if $M_{j}\left(x\right)\;\in \;C_{-1}\left(0,\infty \right)$ for all j=1,…,m, then the convolutional product $M^{<1|m>}\left(x\right)$ with m∈N, is also belong to the space, i.e., $M^{<1|m>}\left(x\right)\in C_{-1}\left(0,\infty \right)$.

Let us define multi-kernel *m*-fold sequential GFI and GFD.

**Definition** **6** (Multi-kernel GF operators)**.** *Let kernel pairs $\left(M_{j},\;K_{j}\right)$ with j=1,…,m belong to the Luchko set $L_{n}$ with m,n∈N.*

*The multi-kernel m-fold sequential GFI is defined as a sequential action of GFIs $I_{\left(M_{j}\right)}^{x}\left[u_{j}\right]$ of AO n with the kernels $M_{j}\left(x\right)$ and j=1,…,m, in the form*

(17) $$I_{\left(M\right)}^{<1|m>,x}\left[u\right]\;f\left(u\right)\;:=\;I_{\left(M_{1}\right)}^{x}\left[x_{2}\right]\;\ldots \;I_{\left(M_{m}\right)}^{x_{m}}\left[u\right]\;f\left(u\right)$$

*with x>0 and $I_{\left(M\right)}^{<1|1>,x}\left[u\right]\;f\left(u\right)\;=\;I_{\left(M_{1}\right)}^{x}\left[u\right]\;f\left(u\right)$, where the GFIs $I_{\left(M_{j}\right)}^{x}\left[u_{j}\right]$ are defined by Equation (5).*

*The multi-kernel m-fold sequential GFD of the Riemann–Liouville and Caputo types are defined as a composition of GFIs $D_{\left(K_{j}\right)}^{x}\left[u_{j}\right]$ of AO n with the kernels $K_{j}\left(x\right)$ and j=1,…,m, in the form*

(18) $$D_{\left(K\right)}^{<1|m>,x}\left[u\right]\;f\left(u\right)\;:=\;D_{\left(K_{1}\right)}^{x}\left[x_{2}\right]\;\ldots \;D_{\left(K_{m}\right)}^{x_{m}}\left[u\right]\;f\left(u\right),$$

(19) $$D_{\left(K\right)}^{<1|m>,x,*}\left[u\right]\;f\left(u\right)\;:=\;D_{\left(K_{1}\right)}^{x,*}\left[x_{2}\right]\;\ldots \;D_{\left(K_{m}\right)}^{x_{m},*}\left[u\right]\;f\left(u\right)$$

*with x>0. For n=1 these operators have the form (6) and (7).*

The following statement describes property of the *m*-fold sequential GFI for multi-kernel case.

**Theorem** **6** (Property of multi-kernel GFI)**.** *Let kernel pairs $\left(M_{j},\;K_{j}\right)$ with j=1,…,m belong to the Luchko set $L_{n}$ with m,n∈N.*

*Then, the multi-kernel GFI (17) of AO can be represented as a GFI with the kernel $M^{<1|m>}\left(x\right)$ in the form*

(20) $$I_{\left(M\right)}^{<1|m>,x}\left[u\right]\;f\left(u\right)\;=\;I_{\left(M^{<1|m>}\right)}^{x}\left[u\right]\;f\left(u\right),$$

*for x>0, if $f\left(x\right)\;\in C_{-1}\left(0,\infty \right)$.*

Theorem 6 is proved in [53].

Using the commutativity and associativity properties of the Laplace convolution, one can obtain that for GFIs the following equality is satisfied

(21) $$I_{\left(M\right)}^{<m|1>,x}\left[u\right]\;f\left(u\right)\;=\;I_{\left(M\right)}^{<1|m>,x}\left[u\right]\;f\left(u\right).$$

It should be emphasized that, in contrast to GFIs, for GFDs the following inequality is satisfied

(22) $$D_{\left(K\right)}^{<m|1>,x}\left[u\right]\;F\left(u\right)\;\neq \;D_{\left(K\right)}^{<1|m>,x}\left[u\right]\;F\left(u\right),$$

in the general case. For the particular case $F\left(x\right)\;\in C_{-1,(M)}^{<1|m>}\left(0,\infty \right)$, the equality is realized.

The following statement describes property of the *m*-fold sequential GFD for multi-kernel case.

**Theorem** **7** (Property of multi-kernel GFD for $C_{-1,(M)}^{<1|m>}\left(0,\infty \right)$)**.** *Let kernel pairs $\left(M_{j},\;K_{j}\right)$ with j=1,…,m belong to the Luchko set $L_{n}$ with m,n∈N.*

*Then, the multi-kernel GFD (18) of AO can be represented as a GFD with the kernel $K^{<1|m>}\left(x\right)$ in the form*

(23) $$D_{\left(K\right)}^{<1|m>,x}\left[u\right]\;F\left(u\right)\;=\;D_{\left(K^{<1|m>}\right)}^{x}\left[u\right]\;F\left(u\right),$$

*for x>0, if $F\left(x\right)\;\in C_{-1,(M)}^{<1|m>}\left(0,\infty \right)$.*

Theorem 7 is proved in [53].

**Definition** **7.** *Let kernel pairs $\left(M_{j},\;K_{j}\right)$ with j=1,…,m belong to the Luchko set $L_{n}$ with m,n∈N, and let a function F(x) can be represented in the form*

(24) $$F\left(x\right)\;=\;I_{\left(M\right)}^{<1|m>,x}\left[u\right]\;f\left(u\right)$$

*for all x>0, where $f\left(x\right)\;\in \;C_{-1}\left(0,\infty \right)$.*

*Then, the set of such functions F(x) is denoted as $C_{-1,(M)}^{<1|m>}\left(0,\infty \right)$.*

The fundamental theorem of the multi-kernel GFC are the following.

**Theorem** **8** (First Fundamental Theorem of multi-kernel GFC of AO)**.** *Let kernel pairs $\left(M_{j},\;K_{j}\right)$ with j=1,…,m belong to the Luchko set $L_{n}$ with m,n∈N.*

*Then, the m-fold sequential GFD (18) of the Riemann–Liouville type is a left inverse operator to the m-fold sequential GFI (17) in the form*

(25) $$D_{\left(K\right)}^{<1|m>,x}\left[u\right]\;I_{\left(M\right)}^{<1|m>,u}\left[w\right]\;f\left(w\right)\;=\;f\left(x\right)$$

*for all x>0, if function f(x) belongs to the space $C_{-1}\left(0,\infty \right)$.*

Theorem 8 is proved in [53].

**Theorem** **9** (Second Fundamental Theorem of multi-kernel GFC of AO for $C_{-1,(M)}^{<1|m>}\left(0,\infty \right)$)**.** *Let kernel pairs $\left(M_{j},\;K_{j}\right)$ with j=1,…,m belong to the Luchko set $L_{n}$ with m,n∈N.*

*Then, the m-fold sequential GFD (18) of the Riemann–Liouville type is a right inverse operator to the m-fold GFI in the form*

(26) $$I_{\left(M\right)}^{<1|m>,x}\left[u\right]\;D_{\left(K\right)}^{<1|m>,u}\left[w\right]\;F\left(w\right)\;=\;F\left(x\right)$$

*for all x>0, if function F(x) belongs to the set $C_{-1,(M)}^{<1|m>}\left(0,\infty \right)$.*

Theorem 9 is proved in [53].

**Remark** **1.** *The second fundamental theorem of multi-kernel GFC of AO, states that the m-fold sequential GFD (18) of AO is also a right inverse operator to the m-fold GFI (17) for the set $C_{-1,(M)}^{<1|m>}\left(0,\infty \right)$.*

*It should be note that for non-local (fractional) PT, it is important to use the second fundamental theorem of GFC in such a form that the multi-kernel GFD is a right inverse operator to multi-kernel GFI.*

### 2.3. Multi-Kernel GFC of AO on Finite Interval [a,b]

The formulation of non-local PT of AO, which was proposed in the previous section for the positive semi-axis (0,∞), can be generalized to intervals including the negative part of the real axis.

**Definition** **8.** *Let a kernel pair (M,K) belong to the Luchko set $L_{n}$, and let a function f(x) belong to the space $C_{-1}\left(0,\infty \right)$. Let −∞<a<b≤∞.*

*Then, the GFI $I_{(M),a+}^{x}$ on the interval (a,b) is defined by the equation*

(27) $$I_{(M),a+}^{x}\left[t\right]f\left(t-a\right)\;:=\;\int_{a}^{x}M\left(x-t\right)\;f\left(t-a\right)\;dt,$$

*where x≥t>a.*

*Then, the GFD $D_{(K),a+}^{x}$ on the interval (a,b) is defined by the equation*

(28) $$D_{(K),a+}^{x}\left[t\right]f\left(t-a\right)\;:=\;\frac{d^{n}}{dx^{n}}\int_{a}^{x}K\left(x-t\right)\;f\left(t-a\right)\;dt,$$

*where x≥t>a.*

**Theorem** **10** (Representation of GF operator on (a,b))**.** *Let a kernel pair (M,K) belong to the Luchko set $L_{n}$, and let a function f(x) belong to the space $C_{-1}\left(0,\infty \right)$. Let −∞<a<b≤∞.*

*Then, the GFI $I_{(M),a+}^{x}$ on the interval (a,b) can be represented as*

(29) $$I_{(M),a+}^{x}\left[t\right]f\left(t-a\right)\;=\;I_{\left(M\right)}^{x-a}\left[u\right]\;f\left(u\right),$$

*where x≥t>a and x−a≥u>0.*

*Then, the GFD $D_{(M),a+}^{x}$ on the interval (a,b) can be represented as*

(30) $$D_{(M),a+}^{x}\left[t\right]f\left(t-a\right)\;=\;D_{\left(M\right)}^{x-a}\left[u\right]\;f\left(u\right),$$

*where x≥t>a and x−a≥u>0.*

Theorem 10 is proved in [53].

Similarly, one can define multi-kernel GF integrals on the finite interval (a,b).

**Definition** **9.** *Let kernel pairs $\left(M_{j},\;K_{j}\right)$ with j=1,…,m belong to the Luchko set $L_{n}$ with n∈N. Let a function f(x) belong to the space $C_{-1}\left(0,\infty \right)$, and let −∞<a<b≤∞.*

*The multi-kernel m-fold sequential GFI on the interval (a,b) is defined as a composition of m GFIs with the kernels $M_{j}\left(x\right)$, j=1,…,m, in the form*

(31) $$\left(I_{(M),a+}^{<1|m>}\;f\right)\left(x\right)\;=\;I_{(M),a+}^{<1|m>,x}\left[u\right]\;f\left(u-a\right)\;:=\;I_{(M_{1}),a+}^{x}\left[x_{2}\right]\;\ldots \;I_{(M_{m}),a+}^{x_{m}}\left[u\right]\;f\left(u-a\right),$$

*where x≥t>a and $I_{(M),a+}^{<1|1>,x}\left[u\right]\;f\left(u-a\right)\;=\;I_{(M_{1}),a+}^{x}\left[u\right]\;f\left(u-a\right)$.*

*The multi-kernel m-fold sequential GFD on the interval (a,b) is defined as a composition of m GFDs with the kernels $K_{j}\left(x\right)$, j=1,…,m, in the form*

(32) $$\left(D_{(K),a+}^{<1|m>}\;f\right)\left(x\right)\;=\;D_{(K),a+}^{<1|m>,x}\left[u\right]\;f\left(u-a\right)\;:=\;D_{(K_{1}),a+}^{x}\left[x_{2}\right]\;\ldots \;D_{(K_{m}),a+}^{x_{m}}\left[u\right]\;f\left(u-a\right),$$

*where x≥t>a and $D_{(K),a+}^{<1|1>,x}\left[u\right]\;f\left(u-a\right)\;=\;D_{(K_{1}),a+}^{x}\left[u\right]\;f\left(u-a\right)$.*

Using Proposition 10, the following theorem is proved in [53].

**Theorem** **11** (Representation of multi-kernel GF operator on (a,b))**.** *Let kernel pairs $\left(M_{j},\;K_{j}\right)$ with j=1,…,m belong to the Luchko set $L_{n}$ with n∈N. Let a function f(x) belong to the space $C_{-1}\left(0,\infty \right)$, and let −∞<a<b≤∞.*

*The multi-kernel m-fold sequential GFI on the interval (a,b) can be represented as*

(33) $$I_{(M),a+}^{<1|m>,x}\left[u\right]\;f\left(u-a\right)\;=\;I_{\left(M\right)}^{<1|m>,x-a}\left[w\right]\;f\left(w\right)$$

*with x>a and $I_{(M),a+}^{<1|1>,x}\left[u\right]\;f\left(u-a\right)\;=\;I_{(M_{1}),a+}^{x}\left[u\right]\;f\left(u-a\right)$, where x≥t>a and x−a≥u>0.*

*The multi-kernel m-fold sequential GFD on the interval (a,b) can be represented as*

(34) $$D_{(K),a+}^{<1|m>,x}\left[u\right]\;f\left(u-a\right)\;=\;D_{\left(K\right)}^{<1|m>,x-a}\left[w\right]\;f\left(w\right).$$

*with x>a and $D_{(K),a+}^{<1|1>,x}\left[u\right]\;f\left(u-a\right)\;=\;D_{(K_{1}),a+}^{x}\left[u\right]\;f\left(u-a\right)$, where x≥t>a and x−a≥u>0.*

Theorem 11 is proved in in [53].

**Remark** **2.** *Equations (33) and (34) express the GFI and GFD on the interval (a,b) in terms of the GFI and GFD by equation for the function $f\left(x\right)\in C_{-1}\left(0,\infty \right)$ that are considered on [0,b−a].*

*As a result of Theorem 11, to consider the GFC on a finite interval (a,b), where −∞<a<b≤∞, one can use the multi-kernel GFC on (0,b−a)⊂(0,∞).*

**Theorem** **12** (First FT of multi-kernel GFC for (a,b))**.** *Let kernel pairs $\left(M_{j},\;K_{j}\right)$ with j=1,…,m belong to the Luchko set $L_{n}$ with m,n∈N.*

*Then, the m-fold sequential GFD (18) of the Riemann–Liouville type is a left inverse operator to the m-fold sequential GFI (17) in the form*

(35) $$D_{(K),a+}^{<1|m>,x}\left[u\right]\;I_{(M),a+}^{<1|m>,u}\left[t\right]\;f\left(t-a\right)\;=\;f\left(x-a\right)$$

*for all x∈(a,b), if function f(x) belongs to the space $C_{-1}\left(0,\infty \right)$.*

Theorem 12 is proved in [53].

**Theorem** **13** (Second FT of multi-kernel GFC for (a,b) and $C_{-1,(M)}^{<1|m>}\left(0,\infty \right)$)**.** *Let kernel pairs $\left(M_{j},\;K_{j}\right)$ with j=1,…,m belong to the Luchko set $L_{n}$ with m,n∈N.*

*Then, the m-fold sequential GFD (18) of the Riemann–Liouville type is a right inverse operator to the m-fold GFI in the form*

(36) $$I_{\left(M,a+\right)}^{<1|m>,x}\left[u\right]\;D_{(K),a+}^{<1|m>,u}\left[t\right]\;F\left(t-a\right)\;=\;F\left(x-a\right)$$

*for all x∈(a,b), if function F(x) belongs to the set $C_{-1,(M)}^{<1|m>}\left(0,\infty \right)$.*

Theorem 13 is proved in [53].

### 2.4. Equations of Multi-Kernel GF Operators of AO

Let us give some statements about multi-kernels GFI and GFD.

**Theorem** **14.** *Let kernel pairs $\left(M_{j},\;K_{j}\right)$ belong to the Luchko set $L_{1}$ for all j=1,…,m with m∈N.*

*Then, kernel pairs $\left(h_{n-1}\;*\;M_{j},\;K_{j}\right)$ belong to the Luchko set $L_{n}$ for all j=1,…,m with m∈N and all n>1, n∈N.*

**Proof.**  Statement of Theorem 14 follows directly from Definition 6 and definition of the Luchko set $L_{n}$. □

**Theorem** **15.** *Let kernel pairs $\left(M_{j},\;K_{j}\right)$ belong to the Luchko set $L_{1}$ for all j=1,…,m with m∈N.*

*Then, the kernels $K^{<1|m>}\left(x\right)$ and $M^{<1|m>}\left(x\right)$ can be represented in the following forms. For all n∈N, the kernels $K^{<1|m>}\left(x\right)$ is*

(37) $$K^{<1|m>}\left(x\right)\;=\;\left(K_{1}\;*\;\ldots \;*\;K_{m}\right)\left(x\right).$$

*For n∈N, the kernels $M^{<1|m>}\left(x\right)$ is*

(38) $$M^{<1|m>}\left(x\right)\;=\;\left\{\begin{array}{cc} \;\left(M_{1}\;*\;\ldots \;*\;M_{m}\right)\left(x\right) & \;n\;=\;1,\\ \left(h_{m(n-1)}\;*\;M_{1}\;*\;\ldots \;*\;M_{m}\right)\left(x\right) & \;n\;\geq 1. \end{array}\right.$$

**Proof.**  Statement of Theorem 15 follows directly from Proposition 14, and Definition 6 of the multi-kernel GFI and GFD of AO and Theorems 6 and 7. □

Let us define a set of kernel pairs for the multi-kernel GFI and GFD of AO.

**Definition** **10.** *Let kernel pairs $\left(M_{j},\;K_{j}\right)$ belong to the Luchko set $L_{1}$ for all j=1,…,m with m∈N.*

*Then, set of kernel pairs $\left(M^{<1|m>},\;K^{<1|m>}\right)$, in which the kernels can be represented in form (37) and (38), is denoted as $L_{n}^{<1|m>}$, where n,m∈N.*

*The kernel pairs $\left(M^{<1|m>},\;K^{<1|m>}\right)$, in which the kernels can be represented in form (37) and (38) with $M_{j}\left(x\right)=M\left(x\right)$ and $K_{j}\left(x\right)=K\left(x\right)$ for all j=1,…,m, are denoted as $L_{n}^{<m>}$.*

**Theorem** **16.** *Let a kernel pair $\left(M^{<1|m>},\;K^{<1|m>}\right)$ belong to the set $L_{n}^{<1|m>}$ such that the kernels can be represented in form (37) and (38), where pairs $\left(M_{j},\;K_{j}\right)$ belong to the Luchko set $L_{1}$ for all j=1,…,m with m∈N.*

*Then, the multi-kernel GFI of AO can be represented by the equations*

(39) $$\begin{array}{c} I_{\left(M\right)}^{<1|m>,x}\left[u\right]\;f\left(u\right)\;=\;\int_{0}^{x}M^{<1|m>}\left(x-u\right)\;f\left(u\right)\;du\;=\;\\ \int_{0}^{x}\left(h_{m(n-1)}\;*\;M_{1}\;*\;\ldots \;*\;M_{m}\right)\left(x-u\right)\;f\left(u\right)\;du, \end{array}$$

*if $f\left(x\right)\in C_{-1}\left(0,\infty \right)$ and n>1. For n=1, the multi-kernel GFI and GFD of AO has the form*

(40) $$I_{\left(M\right)}^{<1|m>,x}\left[u\right]\;f\left(u\right)\;=\;\int_{0}^{x}M^{<1|m>}\left(x-u\right)\;f\left(u\right)\;du\;=\;\int_{0}^{x}\left(M_{1}\;*\;\ldots \;*\;M_{m}\right)\left(x-u\right)\;f\left(u\right)\;du.$$

*Then, the multi-kernel GFD of AO can be represented by the equations*

(41) $$\begin{array}{c} D_{\left(K\right)}^{<1|m>,x}\left[u\right]\;F\left(u\right)\;=\;\frac{d^{nm}}{dx^{nm}}\;\int_{0}^{x}K^{<1|m>}\left(x-u\right)\;F\left(u\right)du\;=\;\\ \frac{d^{nm}}{dx^{nm}}\;\int_{0}^{x}\left(K_{1}\;*\;\ldots \;*\;K_{m}\right)\left(x-u\right)\;F\left(u\right)du, \end{array}$$

*if $F\left(x\right)\in C_{-1,(M)}^{<1|m>}\left(0,\infty \right)$.*

The proof of Theorem 16 is given in [53].

Note that the kernels $M^{<1|m>}\left(x\right)$ are defined for the kernels from the Luchko set $L_{n}$, where the kernel of GFI contains the function ${\left\{1\right\}}^{n-1}=h_{n-1}\left(x\right)$ (see Theorem 1). Therefore, Equation (38) contains the function $h_{m(n-1)}$ since the kernels $M_{j}\left(x\right)$ belong to the Luchko set $L_{1}$.

### 2.5. Examples of Kernel Pairs from Luchko Set ${\;L}_{1}$

Let us define some special function to simplify the notations and calculation.

(42) $$h_{\alpha }\left(x\right)\;:=\;\frac{x^{\alpha -1}}{\Gamma (\alpha )}.$$

(43) $$h_{\alpha ,\beta }\left(x\right)\;:=\;\frac{x^{\alpha -1}}{\Gamma (\alpha )}e^{-\;\beta \;x}.$$

(44) $$\gamma_{\alpha }\left(x\right)\;:=\;\frac{\gamma (\alpha ,x)}{\Gamma (\alpha )},$$

where γ(α,x) is the incomplete gamma function (see Section 9 in [84], pp. 134–142).

(45) $$e_{\alpha ,\beta }\left(x\right)\;:=\;x^{\beta -1}\;E_{\alpha ,\beta }\left[-\;x^{\alpha }\right],$$

where 0<α≤β−1, $E_{\alpha ,\beta }\left[z\right]$ is the two-parameters Mittag–Leffler function (see Section 3 in [85], pp. 17–54, [86] and Section 1.8 in [4], pp. 40–45).

(46) $$\omega_{\alpha }\left(x\right)\;:=\;{\left(\sqrt{x}\right)}^{\alpha -1}\;J_{\alpha -1}\left(2\sqrt{x}\right),$$

where the function $J_{\alpha }\left(u\right)$ is the Bessel function.

(47) $$\phi_{\alpha ,\beta }\left(x\right)\;:=\;x^{\beta -1}\Phi \left(\alpha ,\beta ;-\;x\right),$$

where Φ(α;β;x) is the confluent hypergeometric Kummer function (Section 1.6 in [4], pp. 29–30).

In order for the kernels to describe the operator kernels belonging to the set $L_{1}$, the parameter values in these kernels must be restricted. For example, the parameters of functions (42) and (46) are α∈(0,1); the parameters of functions (45) and (47) are 0<α≤β<1; the parameters of function (43) are α∈(0,1) and β>0. However, when considering sets $L_{n}$, $L_{n}^{<m>}$ and, $L_{n}^{<1|m>}$, the restrictions on the range of values of these parameters change.

Note that functions (42), (43) and (46) belong to the set $C_{-1}\left(0,\infty \right)$, if α>0. Functions (45) and (47) belong to the set $C_{-1}\left(0,\infty \right)$, if β>0.

Let us give examples of kernel pairs $\left(M_{j}\left(x\right),\;K_{j}\left(x\right)\right)$ that belong to the Luchko set $L_{1}$ and have physical dimensions $\left[M_{j}\left(x\right)\right]={\left[x\right]}^{0}$ and $\left[K_{j}\left(x\right)\right]={\left[x\right]}^{-1}$, where j∈N. In these examples, λ>0, $\left[\lambda \right]={\left[x\right]}^{-1}$, and x>0.

- The first kernel pair
  (48) $$M_{a}\left(x\right)\;=\;h_{\alpha }\left(\lambda x\right)\;=\;\frac{{\left(\lambda \;x\right)}^{\alpha -1}}{\Gamma (\alpha )},\;K_{a}\left(x\right)\;=\;\lambda \;h_{1-\alpha }\left(\lambda x\right)\;=\;\frac{\lambda \;{\left(\lambda \;x\right)}^{-\alpha }}{\Gamma (1-\alpha )}.$$
- The second kernel pair
  (49) $$M_{b}\left(x\right)\;=\;h_{\alpha ,\lambda }\left(\lambda \;x\right)\;=\;\frac{{\left(\lambda \;x\right)}^{\alpha -1}}{\Gamma (\alpha )}e^{-\lambda \;x},\;K_{b}\left(x\right)\;=\;\lambda \;h_{1-\alpha ,\lambda }\left(\lambda x\right)\;+\;\lambda \;\gamma_{1-\alpha }\left(\lambda x\right).$$
- The third kernel pair
  (50) $$M_{c}\left(x\right)\;=\;e_{\alpha ,\beta }\left(\lambda \;x\right),\;K_{c}\left(x\right)\;=\;\lambda \;h_{\alpha -\beta ,\lambda }\left(\lambda \;x\right)\;+\;\lambda \;h_{1-\beta }\left(\lambda \;x\right).$$
- The fourth kernel pair
  (51) $$M_{d}\left(x\right)\;=\;\omega_{\alpha }\left(\lambda \;x\right),\;K_{d}\left(x\right)\;=\;\lambda \;i^{\alpha -1}\;\omega_{\alpha }\left(\lambda \;i\;x\right).$$
- The fifth kernel pair
  (52) $$M_{f}\left(x\right)\;=\;\phi_{\alpha ,\beta }\left(\lambda \;x\right),\;K_{e}\left(x\right)\;=\;\frac{\lambda \;\sin(\pi \beta )}{\pi }\;\phi_{\alpha ,1-\beta }\left(\lambda \;x\right),$$

**Remark** **3.** *For other examples of kernel pairs from the Luchko set $L_{1}$, see article [65]. Note that examples can be expanded by using kernel pairs of the form $\left(M_{j,new}=\lambda^{-1}K_{j}\left(x\right),K_{j,new}=\lambda M_{j}\left(x\right)\right)$ for each pair $\left(M_{j}\left(x\right),K_{j}\left(x\right)\right)$ of these examples [65].*

## 3. Nonlocal and GF Probability

### 3.1. Nonlocal (GF) PDF

Let us define a set of non-negative functions that can be used in nonlocal generalization of SPT.

**Definition** **11.** *Let kernel pairs $\left(M_{j}\left(x\right),K_{j}\left(x\right)\right)$ belong to the Luchko set $L_{n}$ for all j=1,…,m, where m∈N, and let a function f(x) can be represented in the form*

(53) $$f\left(x\right)\;=\;I_{\left(K\right)}^{<1|m>,x}\left[u\right]\;\varphi \left(u\right)$$

*for all x>0, where*

(54) $$\varphi \left(x\right)\;\in \;C_{-1}\left(0,\infty \right),$$

*and*

(55) φ(x)≥0,forallx≥0.

*Then, the set of such functions f(x) is denoted as $C_{-1,(K)}^{<1|m>,+}\left(0,\infty \right)$.*

If condition (55) is violated in Definition 11, then the $f\left(x\right)\;\in \;C_{-1,(K)}^{<1|m>}\left(0,\infty \right)$ (see Definition 7).

Let us define the nonlocal and GF PDFs.

**Definition** **12.** *Let kernel pairs $\left(M_{j}\left(x\right),K_{j}\left(x\right)\right)$ belong to the Luchko set $L_{n}$ for all j=1,…,m, where m∈N, and let f(x) satisfy the conditions*

(56) f(x)≥0forallx>0,

(57) $$f\left(x\right)\;\in \;C_{-1,(K)}^{<1|m>}\left(0,\infty \right),$$

*and*

(58) $$I_{\left(M\right)}^{<1|m>,b}\left[u\right]\;f\left(u\right)\;:=\;\lim_{x\to b}\;I_{\left(M\right)}^{<1|m>,x}\left[u\right]\;f\left(u\right)\;=\;1,$$

*where 0<b≤∞.*

*Then, such function f(x) is called the nonlocal PDF. The set of such functions f(x) is denoted as $C_{PDF}^{<1|m>}\left(0,\infty \right)$.*

**Definition** **13.** *Let kernel pairs $\left(M_{j}\left(x\right),K_{j}\left(x\right)\right)$ belong to the Luchko set $L_{n}$ for all j=1,…,m, where m∈N, and let f(x) satisfy the conditions*

(59) $$f\left(x\right)\;\in \;C_{-1,(K)}^{<1|m>,+}\left(0,\infty \right),$$

*and*

(60) $$I_{\left(M\right)}^{<1|m>,b}\left[u\right]\;f\left(u\right)\;:=\;\lim_{x\to b}\;I_{\left(M\right)}^{<1|m>,x}\left[u\right]\;f\left(u\right)\;=\;1$$

*where 0<b≤∞.*

*Then, such function f(x) is called the GF PDF (or complete GF PDF). The set of such functions f(x) is denoted as $C_{PDF}^{<1|m>,+}\left(0,\infty \right)$.*

**Remark** **4.** *It should be noted that the nonlocal and GF PDFs are defined by the pair (f(x),K(x), where the function K(x) describes nonlocality in space. Therefore, these functions should be denoted as $f_{\left(K\right)}\left(x\right)$.*

**Remark** **5.** *Let kernel pairs $\left(M_{j}\left(x\right),K_{j}\left(x\right)\right)$ belong to the Luchko set $L_{n}$ for all j=1,…,m, and let $f\left(x\right)\;\in \;C_{-1,(K)}^{<1|m>,+}\left(0,\infty \right)$ satisfy the condition*

(61) $$I_{\left(M\right)}^{<1|m>,b}\left[u\right]\;f\left(u\right)\;:=\;\lim_{x\to b}\;I_{\left(M\right)}^{<1|m>,x}\left[u\right]\;f\left(u\right)\;<\;\infty ,$$

*where 0<b≤∞.*

*Then, the function*

(62) $$f_{\left(K\right)}\left(x\right)\;=\;\frac{1}{I_{\left(M\right)}^{<1|m>,b}\left[u\right]\;f\left(u\right)}\;f\left(x\right)$$

*is the GF PDF for (0,b).*

Let us prove the non-negativity property of the functions $f\left(x\right)\;\in \;C_{-1,(K)}^{<1|m>,+}\left(0,\infty \right)$.

**Property** **1.** *Let a function f(x) belong to the set $C_{-1,(K)}^{<1|m>,+}\left(0,\infty \right)$, where m∈N.*

*Then, the function f(x) is non-negative*

(63) f(x)≥0

*for all x≥0.*

**Proof.**  Using Definition 11, one can see that function $f\left(x\right)\;\in \;C_{-1,(K)}^{<1|m>,+}\left(0,\infty \right)$ can be represented in the form

(64) $$f\left(x\right)\;=\;I_{\left(K\right)}^{<1|m>,x}\left[u\right]\;\varphi \left(u\right),$$

where φ(x)≥0 for all x>0 and $\varphi \left(x\right)\;\in \;C_{-1}\left(0,\infty \right)$. The proof of Property 1 is based on Definition 6 of the integral $I_{\left(K\right)}^{<1|m>,x}$ and the following statement. If functions $g_{k}\left(x\right)$ and $K_{k}\left(x\right)$ belong to the space $C_{-1}\left(0,\infty \right)$ and the functions $g_{k}\left(x\right)$ and $K_{k}\left(x\right)$ are non-negative functions for all x≥0 ($g_{k}\left(x\right)\geq 0$ and $K_{k}\left(x\right)\geq 0$ for all x≥0), then the function

(65) $$g_{k+1}\left(x\right)\;:=\;I_{\left(K_{k}\right)}^{x}\left[u\right]\;g_{k}\left(u\right)$$

is non-negative function ($g_{k+1}\left(x\right)\geq 0$) for all x≥0. Using $g_{1}\left(x\right)=\varphi \left(x\right)$, $g_{2}\left(x\right)\;:=\;I_{\left(K_{m}\right)}^{x}\left[u\right]\;g_{1}\left(u\right)$ and Equation (65), (17), the repeated application of this statement gives

(66) $$f\left(x\right)\;=\;I_{\left(K\right)}^{<1|m>,x}\left[u\right]\;\varphi \left(u\right)\;:=\;I_{\left(K_{1}\right)}^{x}\left[x_{2}\right]\;\ldots \;I_{\left(K_{m}\right)}^{x_{m}}\left[u\right]\;\varphi \left(u\right)\;\geq \;0$$

for all x>0. As a result, Property 1 was proved. □

As a corollary of Property 1 one can state that GF PDF is non-negative function for all x>0.

Property 1 means that the GF PDFs (or complete GF PDF) are the nonlocal PDFs.

### 3.2. Nonlocal (GF) CDF

Let f(x) belong to the set $C_{-1,(K)}^{<1|m>,+}\left(0,\infty \right)$. Then, one can consider a GF integral of AO of this function in the form

(67) $$F\left(x\right)\;=\;I_{\left(M\right)}^{<1|m>,x}\left[u\right]\;f\left(u\right).$$

Let us assume that

(68) $$F\left(b\right)\;:=\;\lim_{x\to b}\;F\left(x\right)\;<\;\infty ,$$

where x∈(0,b) and 0<b≤∞. Then, using F(x) for the finite interval (0,b), one can consider the function

(69) $$F_{\left(M\right)}\left(x\right)\;=\;\frac{1}{F(b)}\;F\left(x\right)$$

that can be interpreted as a GF CDF.

Let us give definitions of the nonlocal and GF CDFs.

**Definition** **14.** *Let kernel pairs $\left(M_{j}\left(x\right),K_{j}\left(x\right)\right)$ belong to the Luchko set $L_{n}$ for all j=1,…,m, and let F(x) satisfy the conditions*

(70) $$F\left(x\right)\;\in \;C_{-1,(M)}^{<1|m>,+}\left(0,\infty \right),$$

(71) $$\left(D_{\left(K\right)}^{<1|m>,x}F\right)\left(x\right)\;\in \;C_{-1,(K)}^{<1|m>}\left(0,\infty \right),$$

*and*

(72) $$F\left(b\right)\;:=\;\lim_{x\to b}\;F\left(x\right)\;=\;1$$

*where x∈(0,b) and 0<b≤∞.*

*Then, such function F(x) is called the nonlocal CDF. The set of such functions F(x) is denoted as $C_{CDF}^{<1|m>}\left(0,\infty \right)$.*

Note that condition (70) means the existence of the function f(x) such that

(73) $$F\left(x\right)\;=\;I_{\left(M\right)}^{<1|m>,x}\left[u\right]\;f\left(u\right),$$

where f(x)≥0 for all x>0 and $f\left(x\right)\;\in \;C_{-1}\left(0,\infty \right)$. However, this does not assume that $f\left(x\right)\;\in \;C_{-1,(K)}^{<1|m>}\left(0,\infty \right)$. or $f\left(x\right)\;\in \;C_{-1,(K)}^{<1|m>,+}\left(0,\infty \right)$.

Condition (71) is used to have the important property of the nonlocal CDF at x→0+ in the form

(74) $$\lim_{x\to 0+}\;F_{\left(M\right)}\left(x\right)\;=\;0.$$

Let us define a special case of nonlocal CDF.

**Definition** **15.** *Let kernel pairs $\left(M_{j}\left(x\right),K_{j}\left(x\right)\right)$ belong to the Luchko set $L_{n}$ for all j=1,…,m, and let F(x) satisfy the conditions*

(75) $$F\left(x\right)\;\in \;C_{-1,(M)}^{<1|m>}\left(0,\infty \right),$$

(76) $$\left(D_{\left(K\right)}^{<1|m>,x}F\right)\left(x\right)\;\in \;C_{-1,(K)}^{<1|m>,+}\left(0,\infty \right),$$

*and*

(77) $$F\left(b\right)\;:=\;\lim_{x\to b}\;F\left(x\right)\;=\;1$$

*where x∈(0,b) and 0<b≤∞.*

*Then, such function F(x) is called the GF CDF (or complete GF CDF). The set of such functions F(x) is denoted as $C_{CDF}^{<1|m>,+}\left(0,\infty \right)$.*

Please note that the terms in this article have changed slightly from the article [65]. Here, the term “nonlocal” is used instead of “GF” and the term “GF” is used instead of “complete GF” for PDF and CDF.

**Remark** **6.** *In Definition 15 one can use the condition $F\left(x\right)\;\in \;C_{-1,(M)}^{<1|m>}\left(0,\infty \right)$ instead of $F\left(x\right)\;\in \;C_{-1,(M)}^{<1|m>,+}\left(0,\infty \right)$. This is due to the fact that the conditions of Definition 15 are sufficient to obtain the condition $F\left(x\right)\;\in \;C_{-1,(M)}^{<1|m>,+}\left(0,\infty \right)$ as a property of GF CDF (see Property 3 that is proved below).*

**Remark** **7.** *It should be noted that the nonlocal and GF CDFs are defined by the pair (F(x),M(x)), where the function M(x) describes nonlocality in space. Therefore these CDFs should be denoted as $F_{\left(M\right)}\left(x\right)$.*

**Remark** **8.** *It should be noted that the condition $F_{\left(M\right)}\left(x\right)\;\in \;C_{-1,(M)}^{<1|m>,+}\left(0,\infty \right)$ does not lead to non-negativity of the nonlocal probability $P_{\left(M\right)}\left(A\right)$ for all A∈B(Ω), which is defined by the equation*

(78) $$P_{\left(M\right)}\left(x_{1},x_{2}\right]\;:=\;F_{\left(M\right)}\left(x_{2}\right)\;-\;F_{\left(M\right)}\left(x_{1}\right),$$

*where $0\;<\;x_{1}\;<\;x_{2}\;<\;\infty$.*

*To fulfill the non-negativity of the nonlocal probability for all A∈B(Ω), one should impose a stronger condition for the function $F_{\left(M\right)}\left(x\right)$. For details see paper [65].*

*For example, one can consider the following additional condition*

(79) $$F_{\left(M\right)}\left(x\right)\;\in \;C^{1}\left(0,\infty \right),\;\frac{dF_{\left(M\right)}\left(x\right)}{dx}\;\geq \;0\;\left(for\;all\;x>0\right).$$

*To have the GF PDFs, one can consider the condition*

(80) $$\left(D_{\left(K\right)}^{<1|m>,x}F\right)\left(x\right)\;\in \;C_{-1,(K)}^{<1|m>,+}\left(0,\infty \right)$$

*in addition to condition (70).*

**Remark** **9.** *Let us give some remarks for the case (0,∞). In SPT, the PDF $f\left(x\right)\;\in \;C_{-1}\left(0,\infty \right)$ uniquely defines the CDF F(x) by the integration of first order*

(81) $$F\left(x\right)\;=\;\int_{0}^{x}f\left(u\right)\;du.$$

*It can also be state that the standard CDF $F\left(x\right)\;\in \;C_{-1}^{1}\left(0,\infty \right)$. uniquely defines the PDF f(x) by the differentiation of first order*

(82) $$f\left(x\right)\;=\;\frac{d}{dx}F\left(x\right).$$

*The mutual consistency of these concepts is provided by the fundamental theorems of the standard calculus.*

*In nonlocal PT, the PDF cannot uniquely define the CDF. This is due to the fact that for this is also necessary to obtain a function K(x) that describes nonlocality in space.*

*If a pair (f,M) of functions are given, which are interpreted as a generalized PDF and a nonlocality function, then one can specify a function F(x) that will be interpreted as a generalized CDF by the equation F(x)=(M∗f)(x), where ∗ denoted the Laplace convolution. If two pairs $\left(f_{1},\;M_{1}\right)$ and $\left(f_{2},\;M_{2}\right)$ are given, then one can obtain two functions $F_{1}\left(x\right)\;=\;\left(M_{1}\;*\;f_{1}\right)\left(x\right)$ and $F_{2}\left(x\right)\;=\;\left(M_{2}\;*\;f_{2}\right)\left(x\right)$. Obviously, if the functions $f_{1}\left(x\right)$ and $f_{2}\left(x\right)$ are the same for all x>0, then $F_{1}\left(x\right)\;\neq \;F_{2}\left(x\right)$ in general, if $M_{1}\left(x\right)\;\neq \;M_{2}\left(x\right)$.*

*A similar situation for pair (F,K) of functions that are interpreted as a generalized CDF and a nonlocality function. One can specify a function f(x), which will be interpreted as a generalized PDF, by the equation f(x)=(d/dx)(K∗F)(x). If two pairs $\left(F_{1},\;K_{1}\right)$ and $\left(F_{2},\;K_{2}\right)$ are given, then one can obtain two functions $f_{1}\left(x\right)\;=\;\left(d/dx\right)\left(K_{1}\;*\;F_{1}\right)\left(x\right)$ and $f_{2}\left(x\right)\;=\;\left(d/dx\right)\left(K_{2}\;*\;F_{2}\right)\left(x\right)$. Obviously, if the functions $F_{1}\left(x\right)$ and $F_{2}\left(x\right)$ are the same for all x>0, then $f_{1}\left(x\right)\;\neq \;f_{2}\left(x\right)$ in general, if $K_{1}\left(x\right)\;\neq \;K_{2}\left(x\right)$.*

*As a result, in nonlocal PT, mappings of a pair (f,M) of two functions into a function F(x) must be considered, and mappings of a pair (F,K) of two functions into a function f(x) should also be considered. The mutual consistency of these maps and concepts of nonlocal PDF and nonlocal CDF should be provided by the fundamental theorems of the GFC.*

*Since in the SPT the definition of a CDF defines a uniquely probability space $\left(R_{+,0},B\left(R_{+,0}\right),\;P\right)$ (see [87], p. 185, and [88], p. 34), then when defining a probability space in a nonlocal PT, it is necessary to consider functions of nonlocality M(x) in addition to the probability P. Therefore, GF probability space should be defined as $\left(R_{+,0},B\left(R_{+,0}\right),\;P,\;M\right)$.*

### 3.3. GF Probability of AO on Finite Interval [a,b]

The proposed definitions and properties of GF operators on intervals can be used to consider the GF PDFs of AO, the GF CDFs of AO and the GF probability of AO on the finite intervals [a,b] of the real axis R=(−∞,∞), where −∞<a<b<∞.

Let us give definitions of the GF PDFs of AO and the GF CDFs of AO.

One can state that functions f(x) belongs to the set $C_{-1,(K)}^{<1|m>,+}\left(0,\infty \right)$, if the function f(x) can be represented as

(83) $$f\left(x\right)\;=\;I_{\left(K\right)}^{<1|m>,x}\left[u\right]\;\varphi \left(u\right)$$

for all x>0, where

(84) $$\varphi \left(x\right)\;\in \;C_{-1}\left(0,\infty \right),$$

and

(85) φ(x)≥0,forallx≥0.

Then, Equation (83) can be written as

(86) $$f\left(x-a\right)\;=\;I_{\left(K\right)}^{<1|m>,x-a}\left[u\right]\;\varphi \left(u\right)\;=\;I_{(K),a+}^{<1|m>,x}\left[t\right]\;\varphi \left(t-a\right)\left(I_{(K),a+}^{<1|m>}\;\varphi \right)\left(x\right),$$

where the following transformations are used

(87) $$\left(I_{(K),a+}^{<1|m>}\;\varphi \right)\left(x\right)\;=\;I_{(K),a+}^{<1|m>,x}\left[t\right]\;\varphi \left(t-a\right)\;=\;I_{\left(K\right)}^{<1|m>,x-a}\left[u\right]\;\varphi \left(u\right).$$

Let us define a nonlocal (GF) PDF of AO on the finite interval [a,b].

**Definition** **16.** *Let a function f(x) that belongs to the set $C_{-1,(K)}^{<1|m>,+}\left(0,\infty \right)$ satisfy the condition*

(88) $$\left(I_{(M),a+}^{<1|m>}\;f\right)\left(b\right)\;:=\;\lim_{x\to b-}\;\left(I_{(M),a+}^{<1|m>}\;f\right)\left(x\right)\;<\;\infty ,$$

*where −∞<a<b<∞.*

*Then, the function*

(89) $$f_{\left(K\right)}\left(x\right)\;=\;\frac{1}{\left(I_{(M),a+}^{<1|m>}\;f\right)\left(b\right)}\;f\left(x-a\right)$$

*is called the GF PDF (GF PDF) on the interval [a,b].*

Using the function $f\left(x\right)\;\in \;C_{-1,(K)}^{<1|m>,+}\left(0,\infty \right)$, which satisfies condition (88), one can define the function F(x) by the equation

(90) $$F\left(x\right)\;=\;I_{\left(M\right)}^{<1|m>,x}\left[u\right]\;f\left(u\right).$$

Equation (90) can be written as

(91) $$F\left(x-a\right)\;=\;I_{\left(M\right)}^{<1|m>,x-a}\left[u\right]\;f\left(u\right)\;=\;I_{(M),a+}^{<1|m>,x}\left[t\right]\;f\left(t-a\right)\;=\;\left(I_{(M),a+}^{<1|m>}\;f\right)\left(x\right).$$

Then, the function

(92) $$F_{\left(M\right)}\left(x\right)\;=\;\frac{\left(I_{(M),a+}^{<1|m>}\;f\right)\left(x\right)}{\left(I_{(M),a+}^{<1|m>}\;f\right)\left(b\right)}\;=\;\frac{I_{\left(M\right)}^{<1|m>,x-a}\left[u\right]f\left(u\right)}{(I_{\left(M\right)}^{<1|m>,b-a}\left[u\right]\;f\left(u\right)}\;=\;\frac{F(x-a)}{F(b-a)},$$

where x∈(a,b) and −∞<a<b<∞, can be interpreted as a nonlocal (GF) CDF of AO on the finite interval [a,b]. Let us define this GF CDF.

**Definition** **17.** *Let a function f(x) belong to the set $C_{-1,(K)}^{<1|m>,+}\left(0,\infty \right)$, where m∈N and condition (88) is satisfied.*

*Then, the function*

(93) $$\begin{array}{c} F_{\left(M\right)}\left(x\right)\;=\;I_{(M),a+}^{<1|m>,x}\left[u\right]\;f_{\left(K\right)}\left(u\right)\;=\;\frac{1}{\left(I_{(M),a+}^{<1|m>}\;f\right)\left(b\right)}\;I_{(M),a+}^{<1|m>,x}\left[u\right]\;f\left(u-a\right)\;=\;\\ \frac{1}{\left(I_{(M),a+}^{<1|m>}\;f\right)\left(b\right)}\;\left(I_{(M),a+}^{<1|m>}\;f\right)\left(x\right), \end{array}$$

*where x∈(a,b) and −∞<a<b<∞. is called the GF CDF on the interval [a,b].*

Note that in Equation (93), it is used the following representations

(94) $$F\left(x\right)\;=\;\left(I_{(M),a+}^{<1|m>}\;f\right)\left(x\right)\;=\;I_{(M),a+}^{<1|m>,x}\left[t\right]\;f\left(t-a\right)\;=\;I_{\left(M\right)}^{<1|m>,x-a}\left[u\right]\;f\left(u\right),$$

where 0<t<x and 0<u<x−a.

### 3.4. Properties of Nonlocal and GF CDFs

Let us describe some properties of nonlocal and GF CDFs.

**Theorem** **17.** *Let kernel pairs $\left(M_{j}\left(x\right),K_{j}\left(x\right)\right)$ belong to the Luchko set $L_{n}$ for all j=1,…,m, and let a function f(x) be a nonlocal PDF (or let a function f(x) be a GF PDF)*

*Then, the function*

(95) $$F_{\left(M\right)}\left(x\right)\;=\;I_{\left(M\right)}^{<1|m>,x}\left[u\right]\;f_{\left(K\right)}\left(u\right)\;=\;\frac{1}{I_{\left(M\right)}^{<1|m>,b}\left[u\right]\;f\left(u\right)}\;I_{\left(M\right)}^{<1|m>,x}\left[u\right]\;f\left(u\right),$$

*where x∈(0,b) and 0<b≤∞, is the nonlocal CDF (or GF CDF).*

**Proof.**  (0) Equation (95) means that $F_{\left(M\right)}\left(x\right)\;\in \;C_{-1,(M)}^{<1|m>}\left(0,\infty \right)$

(I) Using Definition 12, one can see that the nonlocal PDF $f_{\left(K\right)}\left(x\right)$ is non-negative, i.e., $f_{\left(K\right)}\left(x\right)\;\geq \;0$ for all x>0. Therefore, we obtain $F_{\left(M\right)}\left(x\right)\;\in \;C_{-1,(M)}^{<1|m>,+}\left(0,\infty \right)$. Normalization condition (58) gives condition (72). As a result, function (95) is nonlocal CDF by Definition 14.

(II) Using Definition 13 and Property 15, one can see that the GF PDF $f_{\left(K\right)}\left(x\right)$ is non-negative, i.e., $f_{\left(K\right)}\left(x\right)\;\geq \;0$ for all x>0. Then, the first fundamental theorem of multi-kernel GFC and Equation (95) give

(96) $$\left(D_{\left(K\right)}^{<1|m>,x}F\right)\left(x\right)\;=\;D_{\left(K\right)}^{<1|m>,x}\left[w\right]I_{\left(M\right)}^{<1|m>,w}\left[u\right]\;f_{\left(K\right)}\left(u\right)\;=\;f_{\left(K\right)}\left(x\right).$$

Using Definition 13 and Property 15, one can see that $f_{\left(K\right)}\left(x\right)\;\in \;C_{-1,(K)}^{<1|m>,+}\left(0,\infty \right)$. Therefore $\left(D_{\left(K\right)}^{<1|m>,x}F\right)\left(x\right)\;\in \;C_{-1,(K)}^{<1|m>,+}\left(0,\infty \right)$. Normalization condition (60) gives condition (77). As a result, function (95) is GF CDF by Definition 15. □

The converse theorem for the GF PDF is also true.

**Theorem** **18.** *Let kernel pairs $\left(M_{j}\left(x\right),K_{j}\left(x\right)\right)$ belong to the Luchko set $L_{n}$ for all j=1,…,m, and let a function $F_{\left(M\right)}\left(x\right)$ is the GF CDF.*

*Then, there is a function $f_{\left(K\right)}\left(x\right)$ that is the GF PDF, such that the GF CDF $F_{\left(M\right)}\left(x\right)$ can be represented in the form*

(97) $$F_{\left(M\right)}\left(x\right)\;=\;I_{\left(M\right)}^{<1|m>,x}\left[u\right]\;f_{\left(K\right)}\left(u\right),$$

*where x∈(0,b) and 0<b≤∞.*

**Proof.**  (0) Let us define the function

(98) $$f_{\left(K\right)}\left(x\right)\;=\;D_{\left(K\right)}^{<1|m>,x}\left[u\right]F_{\left(M\right)}\left(u\right).$$

Using second fundamental theorem of GFC for *m*-fold sequential GFD of AO, one can obtain

(99) $$I_{\left(M\right)}^{<1|m>,x}\left[u\right]\;f_{\left(K\right)}\left(u\right)\;=\;I_{\left(M\right)}^{<1|m>,x}\left[u\right]\;D_{\left(K\right)}^{<1|m>,u}F_{\left(M\right)}\left(w\right)\;=\;F_{\left(M\right)})\left(x\right),$$

if $F_{\left(M\right)}\left(x\right)\;\in \;C_{-1,(M)}^{<1|m>}\left(0,\infty \right)$.

(I) If a function $F_{\left(M\right)}\left(x\right)$ is the GF CDF, then

(100) $$\left(D_{\left(K\right)}^{<1|m>,x}F_{\left(M\right)}\right)\left(x\right)\;\in \;C_{-1,(K)}^{<1|m>,+}\left(0,\infty \right).$$

and

(101) $$F_{\left(M\right)})\left(x\right)\;\in \;C_{-1,(M)}^{<1|m>,+}\left(0,\infty \right).$$

Therefore

(102) $$f_{\left(K\right)}\left(x\right)\;\in \;C_{-1,(K)}^{<1|m>,+}\left(0,\infty \right).$$

and using Equation (99), the function $F_{\left(M\right)})\left(x\right)$ can be represented as

(103) $$F_{\left(M\right)})\left(x\right)\;=\;I_{\left(M\right)}^{<1|m>,x}\left[u\right]\;f_{\left(K\right)}\left(u\right).$$

(II) If a function $F_{\left(M\right)}\left(x\right)$ is the nonlocal CDF, then

(104) $$F_{\left(M\right)})\left(x\right)\;\in \;C_{-1,(M)}^{<1|m>,+}\left(0,\infty \right).$$

This condition means that the function $F_{\left(M\right)})\left(x\right)$ can be represented as

(105) $$F_{\left(M\right)})\left(x\right)\;=\;I_{\left(M\right)}^{<1|m>,x}\left[u\right]\;f\left(u\right),$$

where $f\left(x\right)\;\in \;C_{-1}\left(0,\infty \right)$ and f(x)≥0 for all x>0. Using first fundamental theorem of GFC for *m*-fold sequential GFD of AO, one can obtain

(106) $$D_{\left(K\right)}^{<1|m>,x}\left[u\right]\;F_{\left(M\right)}\left(u\right)\;=\;D_{\left(K\right)}^{<1|m>,x}\left[u\right]\;I_{\left(M\right)}^{<1|m>,u}f\left(w\right)\;=\;f\left(x\right),$$

if $f\left(x\right)\;\in \;C_{-1}\left(0,\infty \right)$. Using Equation (99), we obtain

(107) $$f\left(x\right)\;=\;\;f_{\left(K\right)}\left(x\right)\;\geq \;0.$$

In additional, one can state that that

(108) $$D_{\left(K\right)}^{<1|m>,x}\left[u\right]\;F_{\left(M\right)}\left(u\right)\;\in \;C_{-1}\left(0,\infty \right).$$

If a function $F_{\left(M\right)}\left(x\right)$ is the nonlocal CDF, then one can use the property

(109) $$\left(D_{\left(K\right)}^{<1|m>,x}F_{\left(M\right)}\right)\left(x\right)\;\in \;C_{-1,(K)}^{<1|m>}\left(0,\infty \right).$$

Therefore Equations (106) and (109) give

(110) $$f_{\left(K\right)}\left(x\right)\;\in \;C_{-1,(K)}^{<1|m>}\left(0,\infty \right).$$

As a result, nonlocal CDF $F_{\left(M\right)}\left(x\right)$ can be represented as

(111) $$F_{\left(M\right)}\left(x\right)\;=\;I_{\left(M\right)}^{<1|m>,x}\left[u\right]\;f_{\left(K\right)}\left(u\right),$$

where $f_{\left(K\right)}\left(x\right)$ is nonlocal PDF, i.e., $f_{\left(K\right)}\left(x\right)\;\in \;C_{-1,(K)}^{<1|m>}\left(0,\infty \right)$ and $f_{\left(K\right)}\left(x\right)\geq 0$ for all x>0.

As a result, we proved that the nonlocal and GF CDF can be represented in form (97), where $f_{\left(K\right)}\left(x\right)$ that is the nonlocal and GF PDF, respectively. □

The multi-kernel generalization of the following theorem is important for describing properties of the GF CDFs.

**Theorem** **19.** *Let a pair (M(x),K(x)) belong to the Luchko set $L_{1}$.*

*If $f\left(x\right)\in C_{-1,(K)}\left(0,\infty \right)$, then*

(112) $$\lim_{x\to 0+}I_{\left(M\right)}^{x}\left[u\right]\;f\left(u\right)\;=\;0,\;I_{\left(M\right)}^{x}\left[u\right]\;f\left(u\right)\;\in \;C_{-1}^{1}\left(0,\infty \right).$$

*The inverse statement is also satisfied: If conditions (112) are satisfied, then $f\left(x\right)\in C_{-1,(K)}\left(0,\infty \right)$.*

**Proof.**  The statements of this theorem is prove by Luchko in [42], (see comments on p. 9, and Remark 1 on p. 10 of [42]). □

Using the Luchko theorem (Theorem 19) and the properties of functions $f\left(x\right)\in C_{-1,(M)}^{\left(M\right)}\left(0,\infty \right)$, the important properties of the GF CDFs is proved in [65] for the case n=m=1.

Let us prove a generalization of Theorem 19 for multi-kernel GFC of AO.

**Theorem** **20.** *Let kernel pairs $\left(M_{j}\left(x\right),K_{j}\left(x\right)\right)$ belong to the Luchko set $L_{n}$ for all j=1,…,m, where m∈N.*

*If function f(x) belongs to the set $C_{-1,(K)}^{<1|m>}\left(0,\infty \right)$, then*

(113) $$I_{\left(M\right)}^{<1|m>,x}\left[u\right]\;f\left(u\right)\;\in \;C_{-1}^{nm}\left(0,\infty \right),$$

(114) $$\lim_{x\to 0+}\;I_{\left(M\right)}^{<1|m>,x}\left[u\right]\;f\left(u\right)\;=\;0.$$

**Proof.**  To prove Theorem 20, one can use the Holder’s inequality in the following form. Let p,q∈(1,∞) with 1/p+1/q=1 and $f\left(x\right)\in L_{p}\left(a,b\right)$ and $g\left(x\right)\in L_{q}\left(a,b\right)$. Then, the inequality

(115) $${∥f\;g∥}_{1}\;\leq {\;∥f∥}_{p}\;{∥g∥}_{q}$$

holds in the form

(116) $$\int_{a}^{b}\left|f\left(x\right)\;g\left(x\right)\right|\;dx\;\leq \;{\left(\int_{a}^{b}{\left|f\left(x\right)\right|}^{p}\;dx\right)}^{1/p}\;{\left(\int_{a}^{b}{\left|g\left(x\right)\right|}^{q}\;dx\right)}^{1/q}.$$

Function $f\left(x\right)\in C_{-1,(K)}^{<1|m>}\left(0,\infty \right)$ can be represented in the form $f\left(x\right)\;=\;I_{\left(K\right)}^{<1|m>,x}\left[u\right]\;\varphi \left(u\right)$, where $\varphi \left(x\right)\;\in \;C_{-1}\left(0,\infty \right)$. Therefore, the following transformations can be realized:

(117) $$\begin{array}{c} I_{\left(M\right)}^{<1|m>,x}\left[u\right]\;f\left(u\right)\;=\;I_{\left(M\right)}^{<1|m>,x}\left[u\right]\;I_{\left(K\right)}^{<1|m>,u}\left[w\right]\;\varphi \left(w\right)\;=\;\left(\left(M^{<1|m>}\;*\;K^{<1|m>}\right)\;*\;\varphi \right)\left(x\right)\;=\;\\ \left({\left\{1\right\}}^{n\;m}\;*\;\varphi \right)\left(x\right)\;=\;\left(I_{0+}^{n\;m}\;\varphi \right)\left(x\right). \end{array}$$

where

(118) $$\left(I_{0+}^{n\;m}\;\varphi \right)\left(x\right)\;=\;\int_{0}^{x}h_{nm}\left(x-u\right)\;\varphi \left(u\right)\;du\;=\;\int_{0}^{x}\frac{{\left(x-u\right)}^{nm-1}}{\Gamma (mn)}\;\varphi \left(u\right)\;du.$$

Let us estimate the function $\left(I_{0}^{nm}\;\varphi \right)\left(x\right)$ on the interval (0,1] by using the Holder’s inequality in the form

(119) $$\begin{array}{c} |\left(I_{0}^{nm}\;\varphi \right)\left(x\right)\left|\;=\;\right|\int_{0}^{x}h_{nm}\left(x-u\right)\;\varphi \left(u\right)\;du|\;\leq \;\\ \int_{0}^{x}\left|h_{nm}\left(x-u\right)\;\varphi \left(u\right)\right|\;du\;\leq \;{\left(\int_{0}^{x}{\left|h_{nm}\left(x-u\right)\right|}^{p}\;du\right)}^{1/p}\;{\left(\int_{0}^{x}{\left|\varphi \left(u\right)\right|}^{q}\;du\right)}^{1/q}. \end{array}$$

Firstly, one can use the equality

(120) $$\begin{array}{c} \int_{0}^{x}|h_{nm}{\left(x-u\right)|}^{p}\;du\;=\;\frac{1}{\Gamma (nm)}\int_{0}^{x}{\left|\left(x-u\right)\right|}^{p(nm-1)}\;du\;=\;\\ \frac{1}{\Gamma (nm)}\int_{0}^{x}\xi^{p(nm-1)}\;d\xi \;=\;\frac{x^{p(nm-1)+1}}{\left(p(nm-1)+1)\;\Gamma (nm\right)}. \end{array}$$

Secondly, using that $\varphi \left(x\right)\in C_{-1}\left(0,\infty \right)$ can be represented in the form $\varphi \left(x\right)\;=\;x^{\alpha -1}\;\varphi_{1}\left(x\right)$, where $\varphi_{1}\left(x\right)\in C\left(0,\infty \right)$, one can obtain

(121) $$\int_{0}^{x}{\left|\varphi \left(u\right)\right|}^{q}\;du\;=\;\int_{0}^{x}{\left(u^{\alpha -1}\right)}^{q}\;{\left|\varphi_{1}\left(u\right)\right|}^{q}\;du\;=\;C^{q}\;\int_{0}^{x}u^{q(\alpha -1)}\;du\;=\;C^{q}\;\frac{x^{q(\alpha -1)+1}}{q(\alpha -1)+1},$$

where $|\varphi_{1}\left(x\right)|\;\leq \;C$ for all x∈(0,1].

Then, we obtain the equality in the form

(122) $$\begin{array}{c} |\left(I_{0}^{nm}\;\varphi \right)\left(x\right)|\;\leq \;{\left(\int_{0}^{x}{\left|h_{nm}\left(x-u\right)\right|}^{p}\;du\right)}^{1/p}{\left(\int_{0}^{x}{\left|\varphi \left(u\right)\right|}^{q}\;du\right)}^{1/q}\;\leq \;\\ {\left(\frac{x^{p(nm-1)+1}}{\left(p(nm-1)+1)\;\Gamma (nm\right)}\right)}^{1/p}{\left(C^{q}\;\frac{x^{q(\alpha -1)+1}}{q(\alpha -1)+1}\right)}^{1/q}\;=\;\\ C\;{\left(\frac{1}{\left(p(nm-1)+1)\;\Gamma (nm\right)}\right)}^{1/p}\;{\left(\frac{1}{q(\alpha -1)+1}\right)}^{1/q}\;x^{(nm-1)+1/p+(\alpha -1)+1/q}. \end{array}$$

As a result, using 1/p+1/q=1, one can obtain

(123) $$|I_{\left(M\right)}^{<1|m>,x}\left[u\right]\;f\left(u\right)|\;\leq \;C\;{\left(\frac{1}{\left(p(nm-1)+1)\;\Gamma (nm\right)}\right)}^{1/p}\;{\left(\frac{1}{q(\alpha -1)+1}\right)}^{1/q}\;x^{n\;m\;+\;\alpha \;-\;1},$$

where n,m∈N and α>0, i.e., nm+α−1>0. Therefore, inequality (123) gives (114). □

The property of the nonlocal CDF at x→0+ ($F_{\left(M\right)}\left(0+\right)=0$) is a corollary of Theorem 20.

**Property** **2.** *Let kernel pairs $\left(M_{j}\left(x\right),K_{j}\left(x\right)\right)$ belong to the Luchko set $L_{n}$ for all j=1,…,m, and let $F_{\left(M\right)}\left(x\right)$ be a nonlocal CDF.*

*Then, the equation*

(124) $$\lim_{x\to 0+}\;F_{\left(M\right)}\left(x\right)\;=\;0$$

*is satisfied.*

**Proof.**  If F(x) is nonlocal CFD, then F(x) belongs to the set $C_{-1,(M)}^{<1|m>}\left(0,\infty \right)$ such that

(125) $$F\left(x\right)\;=\;I_{\left(M\right)}^{<1|m>,x}\left[u\right]\;f\left(u\right),$$

where function f(x) belongs to the set $C_{-1,(K)}^{<1|m>}\left(0,\infty \right)$. Then, using Theorem 20, one can obtain

(126) $$|F_{\left(M\right)}\left(x\right)|\;\leq \;C\;{\left(\frac{1}{\left(p(nm-1)+1)\Gamma (nm\right)}\right)}^{1/p}\;{\left(\frac{1}{q(\alpha -1)+1}\right)}^{1/q}\;x^{n\;m\;+\;\alpha \;-\;1},$$

where n,m∈N and α>0. Using that nm+α−1>0, inequality (126) gives

(127) $$\lim_{x\to 0+}\;F_{\left(M\right)}\left(x\right)\;=\;0.$$

This ends the proof. □

**Property** **3.** *Let $F_{\left(M\right)}\left(x\right)$ be a GF CDF. Then, the function $F_{\left(M\right)}\left(x\right)$ belongs to the following sets*

(128) $$F_{\left(M\right)}\left(x\right)\;\in \;C_{-1,(M)}^{<1|m>,+}\left(0,\infty \right).$$

**Proof.**  The proof directly follows from the definition of the GF CDF and the definitions of the set $C_{-1,(M)}^{<1|m>,+}\left(0,\infty \right)$, since f(x)≥0 for all x≥0 due to Theorem 18. □

Note that Property 3 means that GF CDF is nonlocal CDF.

**Property** **4.** *Let $F_{\left(M\right)}\left(x\right)$ be a nonlocal or GF CDF. Then, the non-negativity condition*

(129) $$F_{\left(M\right)}\left(x\right)\;\geq \;0$$

*is satisfied for all x>0.*

**Proof.**  For nonlocal CDF, Equation (70) of Definition 14 F(x) satisfies the condition

(130) $$F\left(x\right)\;\in \;C_{-1,(M)}^{<1|m>,+}\left(0,\infty \right).$$

For GF CDFs, Equation (130) is satisfied by Property 3.

Condition (130) means that the function $F\left(x\right)\;\in \;C_{-1,(M)}^{<1|m>,+}\left(0,\infty \right)$ can be represented in the form

(131) $$F\left(x\right)\;=\;I_{\left(M\right)}^{<1|m>,x}\left[u\right]\;f\left(u\right),$$

where f(x)≥0 for all x>0 due to definition of the set $C_{-1,(M)}^{<1|m>,+}\left(0,\infty \right)$.

Then, the proof is based on Definition 6 of the integral $I_{\left(M\right)}^{<1|m>,x}$ and the following statement. If functions $G_{j}\left(x\right)$ and $M_{j}\left(x\right)$ belong to the space $C_{-1}\left(0,\infty \right)$ and the functions $G_{j}\left(x\right)$ and $M_{j}\left(x\right)$ are non-negative functions for all x≥0 ($G_{j}\left(x\right)\geq 0$ and $M_{j}\left(x\right)\geq 0$ for all x≥0), then the function

(132) $$G_{j+1}\left(x\right)\;:=\;I_{\left(M_{j}\right)}^{x}\left[u\right]\;G_{j}\left(u\right)$$

is non-negative function ($G_{k+1}\left(x\right)\geq 0$) for all x≥0. Using $G_{1}\left(x\right)=f\left(x\right)$, $G_{2}\left(x\right)\;:=\;I_{\left(M_{m}\right)}^{x}\left[u\right]\;G_{1}\left(u\right)$ and Equation (132), the repeated application of this statement gives that

(133) $$I_{\left(M\right)}^{<1|m>,x}\left[u\right]\;f\left(u\right)\;:=\;I_{\left(M_{1}\right)}^{x}\left[x_{2}\right]\;\ldots \;I_{\left(M_{m}\right)}^{x_{m}}\left[u\right]\;f\left(u\right)$$

in non-negative for all x≥0. Then, using Equation (131), one can obtain (129) that, proves Property 4. □

**Property** **5.** *Let $F_{\left(M\right)}\left(x\right)$ be a nonlocal or GF CDF. Then, the following condition of non-negativity of the GFD for function $F_{\left(M\right)}\left(x\right)$ in the form*

(134) $$D_{\left(K\right)}^{<1|m>,x}\left[u\right]\;F_{\left(M\right)}\left(u\right)\;\geq \;0$$

*is satisfied for all x>0.*

**Proof.**  Using Theorem 18, one can state that for the GF CDF (and nonlocal CDF) there is a function $f_{\left(K\right)}\left(x\right)\;\in \;C_{-1,(K)}^{<1|m>,+}\left(0,\infty \right)$ (or $f_{\left(K\right)}\left(x\right)\;\in \;C_{-1,(K)}^{<1|m>}\left(0,\infty \right)$ and $f_{\left(K\right)}\left(x\right)\;\geq \;0$ for all x>0), such that the function can be represented in the form

(135) $$F_{\left(M\right)}\left(x\right)\;=\;I_{\left(M\right)}^{<1|m>,x}\left[u\right]\;f_{\left(K\right)}\left(u\right).$$

Using the first fundamental theorem of GFC for *m*-fold sequential GFD of AO, one can obtain

(136) $$D_{\left(K\right)}^{<1|m>,x}\left[u\right]\;F_{\left(M\right)}\left(u\right)\;=\;D_{\left(K\right)}^{<1|m>,x}\left[u\right]\;I_{\left(M\right)}^{<1|m>,u}\left[w\right]\;f_{\left(K\right)}\left(w\right)\;=\;f_{\left(K\right)}\left(x\right),$$

where $f_{\left(K\right)}\left(x\right)\geq 0$ for all x>0, since $f_{\left(K\right)}\left(x\right)\;\in \;C_{-1,(K)}^{<1|m>,+}\left(0,\infty \right)$ (or $f_{\left(K\right)}\left(x\right)\;\in \;C_{-1,(K)}^{<1|m>}\left(0,\infty \right)$ and $f_{\left(K\right)}\left(x\right)\geq 0$ for all x>0). □

**Property** **6.** *Let $F_{\left(M\right)}\left(x\right)$ be a nonlocal or GF CDF.*

*Then, the GF normalization condition*

(137) $$\lim_{x\to b-}\;F_{\left(M\right)}\left(x\right)\;=\;1$$

*is satisfied for all 0<x<b, where 0<b≤∞.*

**Proof.**  Using Equation (69), the GF normalization condition gives

(138) $$\lim_{x\to b-}\;F_{\left(M\right)}\left(x\right)\;=\;\lim_{x\to b-}\frac{1}{F(b)}\;F\left(x\right)\;=\;\frac{1}{F(b)}\;\lim_{x\to b-}\;F\left(x\right)\;=\;\frac{1}{F(b)}\;F\left(b\right)\;=\;1.$$

□

The non-decreasing property of the GF CDF can be described in the following way. Note this property is violated for nonlocal CDF.

**Property** **7.** *Let $F_{\left(M\right)}\left(x\right)\in C_{-1,(M)}^{<1|m>,+}\left(0,\infty \right)$ be a GF CDF such that*

(139) $$F_{\left(M\right)}\left(x\right)\;=\;I_{\left(M\right)}^{<1|m>,x}\left[u\right]\;f_{\left(K\right)}\left(u\right),$$

*and let $f_{\left(K\right)}\left(x\right)\in C_{-1,(K)}^{<1|m>,+}\left(0,\infty \right)$ such that*

(140) $$f_{\left(K\right)}\left(x\right)\;=\;I_{\left(K\right)}^{<1|m>,x}\left[u\right]\;\varphi \left(u\right),$$

*where $\varphi \left(u\right)\in C_{-1}\left(0,\infty \right)$ and φ(x)≥0 for all x>0.*

*Then, the non-decreasing property in the form*

(141) $$\frac{d}{dx}F_{\left(M\right)}\left(x\right)\;\geq \;0$$

*is satisfied for all x>0.*

**Proof.**  Using Equations (139) and (140) and the semi-group property of GF integrals, one can obtain

(142) $$\begin{array}{c} \frac{d}{dx}F_{\left(M\right)}\left(x\right)\;=\;\frac{d}{dx}I_{\left(M\right)}^{<1|m>,x}\left[u\right]\;f_{\left(K\right)}\left(u\right)\;=\;\frac{d}{dx}I_{\left(M\right)}^{<1|m>,x}\left[u\right]\;I_{\left(K\right)}^{<1|m>,u}\left[w\right]\;\varphi \left(w\right)\;=\;\\ \frac{d}{dx}\left({\left\{1\right\}}^{n\;m}\;*\;\varphi \right)\left(x\right)\;=\;\frac{d}{dx}\left(I_{0+}^{n\;m}\;\varphi \right)\left(x\right)\;=\;\left(I_{0+}^{n\;m-1}\;\varphi \right)\left(x\right), \end{array}$$

where n∈N and $\left(I_{0+}^{0}\;\varphi \right)\left(x\right)=\varphi \left(x\right)$. Using that φ(x)≥0 for all x>0 and the fact ${\left(x-u\right)}^{n-1}\geq 0$ for all x>u, one can obtain that the property

(143) $$\left(I_{0+}^{n\;m-1}\;\varphi \right)\left(x\right)\;\geq \;0$$

is satisfied for all x>0. As a result, inequality (143) and Equation (142) give

(144) $$\frac{d}{dx}F_{\left(M\right)}\left(x\right)\;\geq \;0$$

for all x>0. Inequality (144) is the same as inequality (141). □

**Remark** **10.** *Let us note that Property 7 is violated if the condition $F\left(x\right)\;\in \;C_{-1,(M)}^{<1|m>,+}\left(0,\infty \right)$ is used instead of $\left(D_{\left(K\right)}^{<1|m>,x}F\right)\left(x\right)\;\in \;C_{-1,(K)}^{<1|m>,+}\left(0,\infty \right)$. In this case, we have $\left(D_{\left(K\right)}^{<1|m>,x}F\right)\left(x\right)\;\geq \;0$ instead of the inequality (141). As a result, the non-negativity of the nonlocal probability for all A∈B(Ω) is violated [65]. In other words, the non-negativity of the GF PDF is not enough for the GF probability to be non-negative [65].*

The property that describes the behavior of the GF CDFs at zero can be described in the following form.

**Property** **8.** *Let kernel pairs $\left(M_{j}\left(x\right),K_{j}\left(x\right)\right)$ belong to the Luchko set $L_{n}$ for all j=1,…,m.*

*Let a function F(x) be a GF CDF, i.e., F(x) belong to the set $C_{-1,(M)}^{<1|m>}\left(0,\infty \right)$ such that*

(145) $$F\left(x\right)\;=\;I_{\left(M\right)}^{<1|m>,x}\left[u\right]\;f\left(u\right),$$

*where function f(x) belongs to the set $C_{-1,(K)}^{<1|m>}\left(0,\infty \right)$ such that*

(146) $$f\left(u\right)\;=\;I_{\left(K\right)}^{<1|m>,u}\left[w\right]\;\varphi \left(w\right)$$

*with $\varphi \left(x\right)\;\in \;C_{-1}\left(0,\infty \right)$ and φ(x)≥0 for all x>0.*

*Then, the equation*

(147) $$\lim_{x\to 0+}(\frac{d^{k}}{dx^{k}}\;F_{\left(M\right)}\left(x\right))\;=\;0$$

*is satisfied for all k=0,⋯,nm−1, where (nm−1)∈N and $F_{\left(M\right)}\left(x\right)$ is defined by Equation (69). In particular,*

(148) $$\lim_{x\to 0+}\;F_{\left(M\right)}\left(x\right)\;=\;0.$$

**Proof.**  A function $f\left(x\right)\in C_{-1,(K)}^{<1|m>}\left(0,\infty \right)$ can be represented in the form $f\left(x\right)\;=\;I_{\left(K\right)}^{<1|m>,x}\left[u\right]$φ(u), where $\varphi \left(x\right)\;\in \;C_{-1}\left(0,\infty \right)$. Therefore, the following transformations are valid:

(149) $$\begin{array}{c} I_{\left(M\right)}^{<1|m>,x}\left[u\right]\;f\left(u\right)\;=\;I_{\left(M\right)}^{<1|m>,x}\left[u\right]\;I_{\left(K\right)}^{<1|m>,u}\left[w\right]\;\varphi \left(w\right)\;=\;\left(\left(M^{<1|m>}\;*\;K^{<1|m>}\right)\;*\;\varphi \right)\left(x\right)\;=\;\\ \left({\left\{1\right\}}^{n\;m}\;*\;\varphi \right)\left(x\right)\;=\;\left(I_{0+}^{n\;m}\;\varphi \right)\left(x\right). \end{array}$$

Then, one can see that the function

$$F\left(x\right)\;=\;I_{\left(M\right)}^{<1|m>,x}\left[u\right]\;f\left(u\right)\;\in \;C_{-1}^{n\;m}\left(0,\infty \right)$$

has the following property

(150) $$\frac{d^{k}}{dx^{k}}\;F\left(x\right)\;=\;\frac{d^{k}}{dx^{k}}\;\left(I_{0+}^{n\;m}\;\varphi \right)\left(x\right)\;=\;\left(I_{0+}^{n\;m-k}\;\varphi \right)\left(x\right)$$

that is satisfied for all k=0,⋯,(nm−1). Equation (150) is given as Equation (63) in [43], p. 11.

Using the property, according to which for a non-negative continuous function φ(x) on an open interval (a,b), with a=0<b<∞, the following limit exists and is equal to zero

(151) $$\lim_{x\to 0+}\;\int_{0}^{x}\varphi \left(u\right)\;du\;=\;0,$$

where x>0. This statement gives that the equation

(152) $$\lim_{x\to 0+}\;\left(I_{0+}^{n\;m-k}\;\varphi \right)\left(x\right)\;=\;0$$

is satisfied for all k=0,⋯,(nm−1). As a result, Equations (150) and (152) give that the equation

(153) $$\lim_{x\to 0+}\;\frac{d^{k}}{dx^{k}}\;F\left(x\right)\;=\;0$$

is satisfied for all k=0,⋯,m−1, where (nm−1)∈N. Therefore, the function $F_{\left(M\right)}\left(x\right)$ satisfies Equations (147) and (148). □

Note this proof cannot be realized for nonlocal CDFs. The nonlocal CDF satisfies Property 2.

**Property** **9.** *Let kernel pairs $\left(M_{j}\left(x\right),K_{j}\left(x\right)\right)$ belong to the Luchko set $L_{n}$ for all j=1,…,m, and let $F_{\left(M\right)}\left(x\right)$ is nonlocal CDF.*

*Then, the integral non-decreasing property for the function $F_{\left(M\right)}\left(x\right)$ is satisfies in the form*

(154) $$\frac{d}{dx}I_{\left(K\right)}^{<1|m>,x}\left[u\right]\;F_{\left(M\right)}\left(u\right)\;\geq \;0$$

*for all x>0.*

**Proof.**  Using Equation (139), and the semi-group property of GF integrals, one can obtain

(155) $$\begin{array}{c} \frac{d}{dx}I_{\left(K\right)}^{<1|m>,x}\left[u\right]\;F_{\left(M\right)}\left(u\right)\;=\;\frac{d}{dx}I_{\left(K\right)}^{<1|m>,x}\left[u\right]\;I_{\left(M\right)}^{<1|m>,x}\left[u\right]\;f_{\left(K\right)}\left(u\right)\;=\;\\ \frac{d}{dx}\left({\left\{1\right\}}^{n\;m}\;*\;f_{\left(K\right)}\right)\left(x\right)\;=\;\frac{d}{dx}\left(I_{0+}^{n\;m}\;f_{\left(K\right)}\right)\left(x\right)\;=\;\left(I_{0+}^{n\;m-1}\;f_{\left(K\right)}\right)\left(x\right), \end{array}$$

where n∈N and $\left(I_{0+}^{0}\;f_{\left(K\right)}\right)\left(x\right)=f_{\left(K\right)}\left(x\right)$. Using that $f_{\left(K\right)}\left(x\right)\geq 0$ for all x>0 and the fact ${\left(x-u\right)}^{n-1}\geq 0$ for all x>u, one can obtain that the inequality

(156) $$\left(I_{0+}^{n\;m-1}\;f_{\left(K\right)}\right)\left(x\right)\;\geq \;0$$

is satisfied for all x>0. As a result, inequality (156) and Equation (155) give

(157) $$\frac{d}{dx}I_{\left(K\right)}^{<1|m>,x}\left[u\right]\;F_{\left(M\right)}\left(u\right)\;\geq \;0$$

for all x>0. As a result, inequality (154) is proved. □

### 3.5. Nonlocal and GF Probability and Its Properties

Let us note that nonlocal and GF CDFs $F_{\left(M\right)}\left(x\right)$ belong to the set $C_{-1,(M)}^{<1|m>,+}\left(0,\infty \right)$, and the GF derivatives $D_{\left(K\right)}^{<1|m>,x}\left[u\right]\;F_{\left(M\right)}\left(u\right)$ of these functions belong to the set $C_{-1,(M)}^{<1|m>}\left(0,\infty \right)$.

The difference between these functions is determined by the fact that the GF derivatives $D_{\left(K\right)}^{<1|m>,x}\left[u\right]\;F_{\left(M\right)}\left(u\right)$ of the GF CDF belongs to a narrower subset $C_{-1,(M)}^{<1|m>,+}\left(0,\infty \right)$ of the set $C_{-1,(M)}^{<1|m>}\left(0,\infty \right)$. Note that the GF derivatives of nonlocal CFD defines the nonlocal probability functions

(158) $$f_{\left(K\right)}\;=\;D_{\left(K\right)}^{<1|m>,x}\left[u\right]\;F_{\left(M\right)}\left(u\right).$$

Therefore the difference between PDFs is determined by the fact that the GF PDF belongs to a narrower set $C_{-1,(M)}^{<1|m>,+}\left(0,\infty \right)$ than the nonlocal PDF that belongs to the set $C_{-1,(M)}^{<1|m>}\left(0,\infty \right)$. Note that nonlocal PDF and GF PDF are non-negative $f_{\left(K\right)}\geq 0$ for all x>0.

**Definition** **18.** *Let kernel pairs $\left(M_{j}\left(x\right),K_{j}\left(x\right)\right)$ belong to the Luchko set $L_{n}$ for all j=1,…,m, and let $F_{\left(M\right)}\left(x\right)$ be a nonlocal CDF.*

*The set of nonlocal CDF $F_{\left(M\right)}\left(x\right)$ is denoted as $C_{CDF}^{<1|m>}\left(0,\infty \right)$.*

*The set of functions $F_{\left(M\right)}\left(x\right)$ is denoted as $C_{CDF}^{<1|m>,+}\left(0,\infty \right)$, if the following condition is satisfied*

(159) $$D_{\left(K\right)}^{<1|m>,x}\left[u\right]\;F_{\left(M\right)}\left(u\right)\;\in \;C_{-1,(M)}^{<1|m>,+}\left(0,\infty \right).$$

*The set of functions $F_{\left(M\right)}\left(x\right)$ is denoted as $C_{CDF}^{<1|m>,-}\left(0,\infty \right)$, if the following conditions are satisfied*

(160) $$F_{\left(M\right)}\left(u\right)\;\in \;C_{CDF}^{<1|m>}\left(0,\infty \right),$$

(161) $$F_{\left(M\right)}\left(u\right)\;\notin \;C_{CDF}^{<1|m>,+}\left(0,\infty \right).$$

Nonlocal CDF $F_{\left(M\right)}\left(x\right)$ that belongs to the set $C_{CDF}^{<1|m>,+}\left(0,\infty \right)$ is the GF CDF. Note that nonlocal CDF and GF CDF are non-negative $F_{\left(M\right)}\geq 0$ for all x>0.

Let us write down all the properties of the nonlocal and GF CDF that are proved in the previous subsections.

**Proposition** **1.** *Let kernel pairs $\left(M_{j}\left(x\right),K_{j}\left(x\right)\right)$ belong to the Luchko set $L_{n}$ for all j=1,…,m, and let $F_{\left(M\right)}\left(x\right)$ belong to the set $C_{CDF}^{<1|m>}\left(0,\infty \right)$.*

*Then, the following properties of functions $F_{\left(M\right)}\left(x\right)\;\in \;C_{CDF}^{<1|m>}\left(0,\infty \right)$ are satisfied.*

*(1) The existence property of a GF PDF: There is a nonlocal PDF $f_{\left(K\right)}\left(x\right)\in C_{PDF}^{<1|m>}$ such that the nonlocal CDF $F_{\left(M\right)}\left(x\right)$ can be represented in the form*

(162) $$F_{\left(M\right)}\left(x\right)\;=\;I_{\left(M\right)}^{<1|m>,x}\left[u\right]\;f_{\left(K\right)}\left(u\right),$$

*If $F_{\left(M\right)}\left(x\right)$ is a GF CDF, i.e. $F_{\left(M\right)}\left(x\right)\;\in \;C_{CDF}^{<1|m>,+}\left(0,\infty \right)$, then $f_{\left(K\right)}\left(x\right)\in C_{PDF}^{<1|m>,+}$ is used in Equation (162).*

*(2) The property of behavior at zero*

(163) $$\lim_{x\to 0+}\;F_{\left(M\right)}\left(x\right)\;=\;0$$

*is satisfied for all $F_{\left(M\right)}\left(x\right)\;\in \;C_{CDF}^{<1|m>}\left(0,\infty \right)$.*

*(3) The GF normalization property*

(164) $$\lim_{x\to b-}\;F_{\left(M\right)}\left(x\right)\;=\;1$$

*is satisfied for all 0<x<b, where 0<b≤∞.*

*(4) The non-negativity property*

(165) $$F_{\left(M\right)}\left(x\right)\;\geq \;0$$

*is satisfied for all x>0.*

*(5) The non-negativity condition for the GF derivative of the function $F_{\left(M\right)}\left(x\right)$ is satisfies*

(166) $$D_{\left(K\right)}^{<1|m>,x}\left[u\right]\;F_{\left(M\right)}\left(u\right)\;\geq \;0$$

*for all x>0.*

*(6) The integral non-decreasing property for the function $F_{\left(M\right)}\left(x\right)$ is satisfies in the form*

(167) $$\frac{d}{dx}I_{\left(K\right)}^{<1|m>,x}\left[u\right]\;F_{\left(M\right)}\left(u\right)\;\geq \;0$$

*for all x>0.*

*(7) The local non-decreasing property in the form*

(168) $$\frac{d}{dx}F_{\left(M\right)}\left(x\right)\;\geq \;0\;\mathrm{for}\;\mathrm{all}\;\;x>0$$

*is satisfied only if $F_{\left(M\right)}\left(x\right)\;\in \;C_{CDF}^{<1|m>,+}\left(0,\infty \right)$. For $F_{\left(M\right)}\left(x\right)\;\in \;C_{CDF}^{<1|m>,-}\left(0,\infty \right)$ inequality (168) is violated.*

Among the listed properties of nonlocal CDF, the difference between nonlocal and CF CDFs lies in the violation of the local non-decreasing property for a nonlocal CDF. In fact, the local non-decreasing property should be replaced by the nonlocal (integral) non-decreasing property. Note that property (166) can be represented in the form of property (167), if n=m=1 [65]. Therefore the nonlocal (integral) non-decreasing property is described by non-negativity of the GF derivative of nonlocal CDF for the case n=m=1.

**Remark** **11.** *Note that property (166) of the non-negativity condition for the GF derivative of $F_{\left(M\right)}\left(x\right)$ in the limit gives property (168) of the local non-decreasing. In the case n=m=1 and the kernel pair*

(169) $$M\left(x\right)\;=\;h_{\alpha }\left(x\right)\;=\;\frac{x^{\alpha -1}}{\Gamma (\alpha )},\;K\left(x\right)\;=\;h_{1-\alpha }\left(x\right)\;=\;\frac{x^{-\alpha }}{\Gamma (1-\alpha )}$$

*that belongs to the Luchko set $L_{1}$ if α∈(0,1), one can consider the limit*

(170) $$\lim_{x\to 1-}D_{\left(h_{1-\alpha }\right)}^{<1|1>,x}\left[u\right]\;F_{\left(h_{\alpha }\right)}\left(u\right)\;=\;\frac{d}{dx}F_{\left(M\right)}\left(x\right)$$

*to obtain property (168) from inequality (166). Therefore property (166) can be interpreted as a nonlocal analog of the local non-decreasing property (168).*

**Definition** **19.** *Let kernel pairs $\left(M_{j}\left(x\right),K_{j}\left(x\right)\right)$ belong to the Luchko set $L_{n}$ for all j=1,…,m, and let $F_{\left(M\right)}\left(x\right)$ be a nonlocal CDF, i.e., $F_{\left(M\right)}\left(x\right)\in C_{CDF}^{<1|m>}\left(0,\infty \right)$.*

*Then, the nonlocal probability $P_{\left(M\right)}\left(a,b\right]$ is defined by the equations*

(171) $$P_{\left(M\right)}\left(X\leq x\right)\;=\;P_{\left(M\right)}\left(0,x\right]\;=\;F_{\left(M\right)}\left(x\right),$$

(172) $$P_{\left(M\right)}\left(a,b\right]\;=\;P\left(a<X\leq b\right)\;=\;F_{\left(M\right)}\left(b\right)\;-\;F_{\left(M\right)}\left(a\right),$$

*if 0≤a<b<∞, and*

(173) $$P_{\left(M\right)}\left(a,\infty \right)\;=\;1\;-\;F_{\left(M\right)}\left(a\right),$$

*if 0≤a<b=∞.*

*If $F_{\left(M\right)}\left(x\right)\;\in \;C_{CDF}^{<1|m>,+}\left(0,\infty \right)$, then the nonlocal probability $P_{\left(M\right)}\left(a,b\right]$ is called the GF probability.*

**Remark** **12.** *It should be emphasized that the nonlocal CDFs are not non-decreasing (in the standard sense) for all x>0, in the general case. Only the GF derivative of the function $F_{\left(M\right)}\left(x\right)$ is non-negative. The first order derivative of this function must not be nonnegative for all x>0. This means that the function $F_{\left(M\right)}\left(x\right)$ can be decreasing on some intervals. For nonlocal CDF $F_{\left(M\right)}\left(x\right)\in C_{CDF}^{<1|m>}\left(0,\infty \right)$, non-decreasing function in the standard sense is realized for all x>0 only in nonlocal (integral) form for the GF integral $I_{\left(K\right)}^{<1|m>,x}\left[u\right]\;F_{\left(M\right)}\left(u\right)$ since*

(174) $$\frac{d}{dx}\;I_{\left(K\right)}^{<1|m>,x}\left[u\right]\;F_{\left(M\right)}\left(u\right)\;\geq \;0.$$

*For nonlocal CDF $F_{\left(M\right)}\left(x\right)\in C_{CDF}^{<1|m>,-}\left(0,\infty \right)$, there is such an interval $\left(a,b\right]\subset R_{+}$ that the first-order derivative of the function $F_{\left(M\right)}\left(x\right)$ is negative. Then, on this interval the function $F_{\left(M\right)}\left(x\right)$ decreases in the standard sense, and*

(175) $$F_{\left(M\right)}\left(b\right)\;<\;F_{\left(M\right)}\left(a\right),$$

*for b>a≥0, As a result, the nonlocal probability $P_{\left(M\right)}\left(a,b\right]$ can be negative*

(176) $$P_{\left(M\right)}\left(a,b\right]\;=\;F_{\left(M\right)}\left(b\right)\;-\;F_{\left(M\right)}\left(a\right)\;\leq \;0.$$

*At the same time, the nonlocal (integral) non-decreasing property*

(177) $$I_{\left(K\right)}^{<1|m>,a}\left[x\right]\;F_{\left(M\right)}\left(x\right)\;<\;I_{\left(K\right)}^{<1|m>,b}\left[x\right]\;F_{\left(M\right)}\left(x\right)$$

*is satisfied and*

(178) $$I_{\left(K\right)}^{<1|m>,[a,b]}\left[x\right]\;P_{\left(M\right)}\left(\left(0,x\right]\right)\;\geq \;0$$

*for every (a,b]⊂(0,∞), since*

(179) $$I_{\left(K\right)}^{<1|m>,[a,b]}\left[x\right]\;P_{\left(M\right)}\left(0,x\right]\;=\;I_{\left(K\right)}^{<1|m>,b}\left[x\right]\;P_{\left(M\right)}\left(0,x\right]\;-\;I_{\left(K\right)}^{<1|m>,a}\left[x\right]\;P_{\left(M\right)}\left(0,x\right]\;\geq \;0,$$

*where $P_{\left(M\right)}\left(0,x\right]=F_{\left(M\right)}\left(x\right)$.*

In general, it is important to study not only the general case, in which the nonlocal probability on the interval can be negative, but also the special case, when the GF probability on the interval is non-negative.

**Proposition** **2.** *Let kernel pairs $\left(M_{j}\left(x\right),K_{j}\left(x\right)\right)$ belong to the Luchko set $L_{n}$ for all j=1,…,m, and let $F_{\left(M\right)}\left(x\right)$ be a nonlocal CDF, i.e., $F_{\left(M\right)}\left(x\right)\in C_{CDF}^{<1|m>}\left(0,\infty \right)$.*

*Then, the nonlocal probability*

(180) $$P_{\left(M\right)}\left(a,b\right]\;=\;F_{\left(M\right)}\left(b\right)\;-\;F_{\left(M\right)}\left(a\right),$$

*where b>a≥0, satisfies the following standard properties.*

*Let $A_{k}$, k∈N be intervals such that $A_{k}=\left(a_{k},b_{k}\right]$, where $0\;\leq \;a_{k}\;<\;b_{k}\;<\;\infty$. Then, the following properties of the nonlocal probability density are satisfied.*

*(1) The non-negativity property*

(181) $$P_{\left(M\right)}\left(A_{k}\right)\;\geq \;0$$

*is satisfied for every $A_{k}$ only if $F_{\left(M\right)}\left(x\right)\;\in \;C_{CDF}^{<1|m>,+}\left(0,\infty \right)$. Note that $P_{\left(M\right)}\left(0,x\right]\;\geq \;0$ for all x>0 for nonlocal CDF $F_{\left(M\right)}\left(x\right)\;\in \;C_{CDF}^{<1|m>}\left(0,\infty \right)$.*

*(2) The normalization property*

(182) $$P_{\left(M\right)}\left(\left(0,\infty \right)\right)\;=\;1$$

*for all $F_{\left(M\right)}\left(x\right)\;\in \;C_{CDF}^{<1|m>}\left(0,\infty \right)$.*

*(3) If $A_{k}\subset A_{j}$, then*

(183) $$P_{\left(M\right)}\left(A_{k}\right)\;\leq \;P_{\left(M\right)}\left(A_{j}\right)$$

*is satisfied for every $A_{k}$ and $A_{j}$ only if $F_{\left(M\right)}\left(x\right)\;\in \;C_{CDF}^{<1|m>,+}\left(0,\infty \right)$.*

*(4) If $A_{k}\cap A_{j}=⌀$, then*

(184) $$P_{\left(M\right)}\left(A_{k}\cup A_{j}\right)\;=\;P_{\left(M\right)}\left(A_{k}\right)\;+\;P_{\left(M\right)}\left(A_{j}\right)$$

*for all $F_{\left(M\right)}\left(x\right)\;\in \;C_{CDF}^{<1|m>}\left(0,\infty \right)$.*

*(5) If $A_{k}\cap A_{j}\neq ⌀$, then*

(185) $$P_{\left(M\right)}\left(A_{k}\cup A_{j}\right)\;=\;P_{\left(M\right)}\left(A_{k}\right)\;+\;P_{\left(M\right)}\left(A_{j}\right)\;-\;P_{\left(M\right)}\left(A_{k}\cap A_{j}\right)$$

*for all $F_{\left(M\right)}\left(x\right)\;\in \;C_{CDF}^{<1|m>}\left(0,\infty \right)$.*

*(6) For every $A_{k}$ and $A_{j}$,*

(186) $$P_{\left(M\right)}\left(A_{k}\cup A_{j}\right)\;\leq \;P_{\left(M\right)}\left(A_{k}\right)\;+\;P_{\left(M\right)}\left(A_{j}\right)$$

*is satisfied for every $A_{k}$ and $A_{j}$ only if $F_{\left(M\right)}\left(x\right)\;\in \;C_{CDF}^{<1|m>,+}\left(0,\infty \right)$.*

**Proof.**  The proof of these properties follows directly from the properties of the nonlocal CDF and Equation (180) that defines the nonlocal probability.

Note that the properties of nonlocal probability for the case m=n=1 are described in paper [65]. Note that in present paper the terms are slightly different from paper [65]. Here, the term “nonlocal” is used instead of “GF” and the term “GF” is used instead of “complete GF” for PDF and CDF. □

**Remark** **13.** *It should be noted that for nonlocal PDFs from a set $C_{PDF}^{<1|m>,-}\left(0,\infty \right)$, the nonlocal probability can be negative for some intervals. However, the nonlocal probability is non-negative for all intervals (0,x],*

(187) $$P_{\left(M\right)}\left(0,x\right]\;\geq \;0\;for\;all\;x>0.$$

**Remark** **14.** *The negativity of the nonlocal probability on some intervals can be interpreted by the fact that nonlocality affects the change in the probability density. This influence leads to the fact that the CDFs may decrease in some regions. Such influence of nonlocality is in some sense similar to the behavior of the Wigner distribution function in quantum statistical mechanics [89,90] and some non-Kolmogorov probability models [91,92,93,94]. This property of nonlocality in the nonlocal PT should not be excluded from consideration. Therefore, one should not limit oneself only to the consideration of the sets $C_{PDF}^{<1|m>,+}\left(0,\infty \right)$ and $C_{CDF}^{<1|m>,+}\left(0,\infty \right)$. It is important to investigate sets $C_{PDF}^{<1|m>}\left(0,\infty \right)$ and $C_{CDF}^{<1|m>}\left(0,\infty \right)$.*

**Remark** **15.** *Theory of nonlocal probability, which is defined by using nonlocal CDFs $F_{\left(M\right)}\left(x\right)$ that belong to the set $C_{CDF}^{<1|m>}\left(0,\infty \right)$ or $C_{PDF}^{<1|m>,-}\left(0,\infty \right)$, can be considered as one of non-Kolmogorov probability models. The nonlocal probability, which is defined by using GF CDFs $F_{\left(M\right)}$ that belong to the set $C_{CDF}^{<1|m>,+}\left(0,\infty \right)$, can be considered as one of special case of Kolmogorov probability models.*

Note that the nonlocal PT cannot be reduced to a SPT that uses the pairs of classical PDFs and CDFs. This statement is similar to the statement that GFC cannot be reduced to the standard calculus of integrals and derivatives of integer orders.

### 3.6. From CDF to Probability Space

First consider the standard probability (measurable) space (R,B(R),P) on the real line R, where B(R) is the system of Borel sets on R, and *P* is a standard probability measure [87,88].

Let us give a definition of the standard CDF (CDF).

**Definition** **20.** *Let (R,B(R)) be the real line R, with the system B(R) of Borel sets. Let P=P(A) be a probability defined on the Borel subset A of the real line R. If A=(−∞,x], then*

(188) F(x)=P(−∞,x]

*is called the CDF.*

The following theorem describes characteristic properties of standard CDFs.

**Theorem** **21.** *Let F(x) be a CDF of a random variable X.*

*Then, F(x) satisfies the following properties:*

*I. Right-continuity: F(x) is continuous function on the right and has a limit on the left at each x∈R.*

(189) $$\lim_{x\to x_{0}+}\;F\left(x\right)\;=\;F\left(x_{0}\right).$$

*II. Monotonicity: F(x) is non-decreasing function. If $-\infty \leq x_{1}<x_{2}\leq \infty$, then $F\left(x_{1}\right)\;\leq \;F\left(x_{2}\right)$.*

*III. Behavior at interval boundaries*

(190) $$\lim_{x\to -\infty }\;F\left(x\right)\;=\;0,$$

(191) $$\lim_{x\to +\infty }\;F\left(x\right)\;=\;1.$$

Theorem 21 is proved in [87], p. 185, and in book [88], p. 34.

One can formulate a theorem inverse to Theorem 21. The inverse theorem shows what properties a function must have in order to be a standard CDF.

**Theorem** **22.** *Let F=F(x) be a function on the real line R, which satisfies conditions I, II, and III.*

*Then, there exists a unique probability space (R,B(R),P) and a random variable X such that*

(192) P(X≤x)=F(x),

(193) $$P\left(x_{1}<X\leq x_{2}\right)\;=\;P\left(x_{1},x_{2}\right]\;=\;F\left(x_{2}\right)\;-\;F\left(x_{1}\right)$$

*for all $x_{1}$, $x_{2}$ such that $-\infty \leq x_{1}<x_{2}<\infty$.*

Theorem 22 is proved in book [88], p. 35, as Theorem 3.2.1, and it is also described in [87], p. 185, as Theorem 1.

As a result, one can state that any function F(x) on the real line R, which satisfies conditions I, II, and III, is a standard CDF. Theorem 22 states that there is a one-to-one correspondence between standard probability measures P(x) on (R,B(R)) and standard CDFs F(x) on the real line R. The measure P(x) constructed from the CDF F(x) is usually called the Lebesgue-Stieltjes probability measure corresponding to the CDF F(x) [87], p. 187.

The correspondence between standard probability measures P(a,b] and standard CDFs F(x) established by the equation P(a,b]=F(b)−F(a) makes it possible to construct various probability measures and standard probability spaces by specifying the corresponding CDFs [87], p. 188.

One can state that this statement and Theorem 22 can be extended to the nonlocal case.

### 3.7. From GF CDF to GF Probability Space

Let us consider an extension of Theorem 22 to the nonlocal case. One can state that the one-to-one correspondence between GF probability $P_{\left(M\right)}\left(x\right)$ and GF CDFs $F_{\left(M\right)}\left(x\right)$ can be proved.

Let us first formulate the theorem about restriction on the GF CDF to perform standard properties I, II, and III.

**Theorem** **23.** *Let F=F(x) be a function on the non-negative semi-axis $R_{+,0}=\left[0,\infty \right)$, which satisfies the following conditions:*

*N1. F(x) belongs to the space $C_{-1,(M)}^{<1|m>,+}\left(0,\infty \right)$.*

*N2. Monotonicity: F(x) is non-decreasing function:*

(194) $$\frac{d}{dx}F\left(x\right)\;\geq \;0.$$

*N3a. Behavior on the left boundary of the interval*

(195) $$\lim_{x\to 0+}\;F\left(x\right)\;=\;0.$$

*N3b. Behavior on the right boundary of the interval*

(196) $$\lim_{x\to \infty }\;F\left(x\right)\;=\;1.$$

*Then, the function F=F(x) satisfies the conditions I, II, and III.*

**Remark** **16.** *Let us note the condition N1 cannot give Condition N2. If the Condition $\left(D_{\left(K\right)}^{<1|m>,x}F\right)\left(x\right)$$\;\in \;C_{-1,(K)}^{<1|m>,+}\left(0,\infty \right)$ is used in addition to the Condition N1, then Condition N2 is satisfied (see Property 4).*

Theorem 23 leads to the following statements.

**Theorem** **24.** *Let $F=F_{\left(M\right)}\left(x\right)$ be a GF CDF on semi-axis $R_{+,0}=\left[0,\infty \right)$.*

*Then, the properties N1, N2, N3 are satisfied.*

Theorem 23 follows from Theorem 22, if we additionally take into account nonlocality in space. Therefore the nonlocal probability space should be considered as $\left(R_{+,0},B\left(R\right),\;P,\;M\right)$, where M(x) is the function that describes nonlocality in space.

**Definition** **21.** *The GF probability space $\left(R_{+,0},B\left(R_{+,0}\right),\;P,\;M\right)$ is the following quartet:*

*(1) The semi-axis $R_{+,0}$ of the real line R.*

*(2) The system $B(R_{+,0})$ of Borel sets on $R_{+,0}$.*

*(3) The probability P(A) for all $A\;\in \;B(R_{+,0})$.*

*(4) The function M(x) of nonlocality in space, for which there is such a function K(x) that the pair (M(x),K(x)) belongs to the set $L_{n}$ or $L_{n}^{<1|m>}$.*

**Remark** **17.** *The standard probability space $\left(R_{+,0},B\left(R\right),P\right)$ can be considered as a limit particular case, in which the Heaviside step function H(x) is considered as a function M(x), and the Dirac delta-function δ(x) is considered as the function K(x).*

(197) $$\left(R_{+,0},\;B\left(R\right),\;P\right)\;=\;\left(R_{+,0},\;B\left(R\right),\;P,\;H\right).$$

*It should be emphasized that the pair of the functions H(x) and δ(x) do not belong to the sets $L_{n}$ or $L_{n}^{<1|m>}$. This limit can be derived by using $M\left(x\right)\;=\;h_{\alpha }\left(x\right)$ with α∈(0,1) at α→1−.*

The following statement describes the one-to-one correspondence between GF probability $P_{\left(M\right)}\left(x\right)$ and GF CDFs $F_{\left(M\right)}\left(x\right)$.

**Theorem** **25.** *Let $F=F_{\left(M\right)}\left(x\right)$ be a GF CDF on $R_{+,0}$.*

*Then, there exists a unique GF probability space $\left(R_{+,0},B\left(R_{+,0}\right),\;P,\;M\right)$ and a random variable X such that*

(198) $$P_{\left(M\right)}\left(X\leq x\right)\;=\;F_{\left(M\right)}\left(x\right),$$

(199) $$P\left(a<X\leq b\right)\;=\;P\left(a,b\right]\;=\;F_{\left(M\right)}\left(b\right)\;-\;F_{\left(M\right)}\left(a\right)$$

*for all a, b such that 0≤a<b<∞.*

Similarly, one can consider the relationship between the GF probability and the GF CDF on a finite interval. For GF probability of finite intervals [a,b], one can have the following definition.

**Definition** **22.** *Let Ω=[a,b] be a finite interval on the real line, where −∞<a<b<∞.*

*Then, the GF probability space (Ω,B(Ω),P,M) is the following quartet:*

*(1) The set Ω⊂R of the real line R.*

*(2) The system B(Ω) of Borel sets on the set Ω.*

*(3) The probability measure P(A) for all A∈B(Ω).*

*(4) The function M(x) of nonlocality in space, for which there is such a function K(x) that their pair (M(x),K(x)) belongs to the set $L_{n}$ or $L_{n}^{<1|m>}$.*

Let us give the following statement about the properties of a function necessary to satisfy conditions I, II, and III for finite intervals.

**Theorem** **26.** *Let [a,b], where −∞<a<b<∞, be a finite interval of the real axis R=(−∞,∞).*

*Let $F=F_{\left(M\right)}\left(x\right)$ be a GF CDF of AO on the interval Ω=[a,b], where −∞<a<b<∞.*

*Then, the following conditions for the function $F=F_{\left(M\right)}\left(x\right)$ are satisfied:*

*A1. $F_{\left(M\right)}\left(x\right)$ belongs to the space $C^{n}\left(a,b\right)$.*

*A2. Monotonicity: F(x) is non-decreasing function:*

(200) $$\frac{d}{dx}F_{\left(M\right)}\left(x\right)\;\geq \;0.$$

*A3a. Behavior on the left boundary of the interval*

(201) $$\lim_{x\to a+}\;F_{\left(M\right)}\left(x\right)\;=\;0.$$

*A3b. Behavior on the right boundary of the interval*

(202) $$\lim_{x\to b-}\;F_{\left(M\right)}\left(x\right)\;=\;1.$$

For GF probability of finite intervals [a,b], Proposition 26 leads to the following statement.

**Theorem** **27.** *Let $F=F_{\left(M\right)}\left(x\right)$ be a GF CDF of AO on the interval Ω=[a,b], where −∞<a<b<∞.*

*Then, there exists a unique GF probability space (Ω,B(Ω),P,M) and a random variable X such that*

(203) $$P_{\left(M\right)}\left(X\leq x\right)\;=\;F_{\left(M\right)}\left(x\right),$$

(204) $$P_{\left(M\right)}\left(c<X\leq d\right)\;=\;P_{\left(M\right)}\left(c,d\right]\;=\;F_{\left(M\right)}\left(d\right)\;-\;F_{\left(M\right)}\left(c\right)$$

*for all c, d such that a≤c<d≤b.*

Theorems 24 and 27 state that the correspondence between the GF probability $P_{\left(M\right)}\left(a,b\right]$ and GF CDFs $F_{\left(M\right)}\left(x\right)$, makes it possible to construct GF probability and GF probability spaces by using the GF CDFs.

### 3.8. Remarks about GF Probability and GF Probability Space

In connection with Theorems 25 and 27, it should be additionally discussed the difference between the GF probability space differ from the standard probability space.

Note that GF PT is different from the SPT, as well as the GFC of integrals and derivatives of AO differ from the standard calculus of derivatives and integrals of the integer orders.

**Remark** **18.** *In the generalization of the SPT to nonlocal PT, the following fact should be taken into account. The SPT uses a pair (F(x),f(x)) of mutually interconnected concepts (functions), namely the CDF (CDF) and the PDF (PDF). The mutual relations of these functions are described by the equations*

(205) $$f\left(x\right)\;=\;\frac{dF(x)}{dx},\;F\left(x\right)\;=\;\int_{0}^{x}f\left(x\right)\;dx,$$

*where f(x)∈C(0,∞) and $F\left(x\right)\;\in \;C^{1}\left(0,\infty \right)$, for example. The mutual consistence of these concepts is based on the fundamental theorems of standard calculus.*

*In this regard, one can use two ways to definitions of probability through one of these functions that are almost equivalent to a wide class of functions. For nonlocal case, it is necessary to additionally define nonlocality in space.*

*In nonlocal PT, it should be used two pair (F(x),f(x)) and (M(x),K(x)) of mutually interconnected concepts [65,80]. In addition to the PDF, a corresponding function of nonlocality K(x) should be considered. In addition to the CDF (CDF), a corresponding function of nonlocality M(x) should be taken into account. Therefore, one should use the notations $f_{\left(K\right)}\left(x\right)$ and $F_{\left(M\right)}$. In the nonlocal theory, the mutual relations of these functions are determined by the GF fundamental theorems of GFC. These theorems lead not only to the interconnection of the function $f_{\left(K\right)}\left(x\right)$ and $F_{\left(M\right)}\left(x\right)$, but also to the conditions for their functions of nonlocality K(x) and M(x) that can be described by Sonin conditions or by the Luchko conditions. In the GFC, the pair of functions (M,K) should belong to the Luchko set $L_{n}$. In the multi-kernel GFC of AO, the pair of functions (M,K) should belong to the set $L_{n}^{<1|m>}$.*

*As a result, to define nonlocal probability space and GF probability, it is necessary to include functions of nonlocality in definitions of the GF probability spaces. Therefore, the GF probability $P_{\left(M\right)}$ is defined by the pair of a distribution function F(x) and function of nonlocality M(x). This pair is represented as a GF CDF of by the notation $F_{\left(M\right)}\left(x\right)$. The GF probability space should be defined as the quaternary (Ω,B(Ω),P,M) instead of (Ω,B(Ω),P). For simplification, it can be denoted as $\left(\Omega ,\;B\left(\Omega \right),\;P_{\left(M\right)}\right)$. Note that the use of different functions (M(x),K(x)) leads to various probability spaces (Ω,B(Ω),P), since P depends on the function M that defines the nonlocality in space (see also Remark 9).*

*One can state that standard (local) theory corresponds to the limit case, where M(x) and K(x), which belong to the set $L_{1}$, are represented by the Heaviside step function and the Dirac delta function, respectively. Note that these functions themselves do not belong to the Luchko set $L_{1}$.*

**Remark** **19.** *To illustrate nonlocal properties of the GF probability, one can consider a function f(x) that can be presented as*

(206) $$f\left(x\right)\;=\;\left\{\begin{array}{cc} f_{1}\left(x\right) & \;x\;\in \;(0,a],\\ f_{2}\left(x\right) & \;x\;\in \;(a,b]. \end{array}\right.$$

*In SPT, the probability P(a,b] does not depend on the behavior of the PDF f(x) at x<a, since*

(207) $$P\left(a,b\right]\;=\;F\left(b\right)\;-\;F\left(a\right)\;=\;\int_{0}^{b}f\left(x\right)\;dx\;-\;\int_{0}^{a}f\left(x\right)\;dx\;=\;\int_{a}^{b}f\left(x\right)\;dx\;=\;\int_{a}^{b}f_{2}\left(x\right)\;dx.$$

*In nonlocal PT, the GF probability $P_{\left(M\right)}\left(a,b\right]$ depends on the behavior of the PDF $f_{\left(K\right)}\left(x\right)=f\left(x\right)$ at x<a, since*

(208) $$\begin{array}{c} P_{\left(M\right)}\left(a,b\right]\;=\;F_{\left(M\right)}\left(b\right)\;-\;F_{\left(M\right)}\left(a\right)\;=\;\int_{0}^{b}M\left(b\;-\;x\right)\;f\left(x\right)\;dx\;-\;\int_{0}^{a}M\left(a\;-\;x\right)\;f\left(x\right)\;dx\;=\;\\ \int_{a}^{b}M\left(b\;-\;x\right)\;f_{2}\left(x\right)\;dx\;+\;\int_{0}^{a}M\left(b\;-\;x\right)\;f_{1}\left(x\right)\;dx\;-\;\int_{0}^{a}M\left(a\;-\;x\right)\;f_{1}\left(x\right)\;dx\;=\;\\ \int_{a}^{b}M\left(b\;-\;x\right)\;f_{2}\left(x\right)\;dx\;+\;\int_{0}^{a}\left(M\left(b\;-\;x\right)\;-\;M\left(a\;-\;x\right)\right)\;f_{1}\left(x\right)\;dx, \end{array}$$

*where M(b−x)−M(a−x)≠0 in the general case.*

*As a result, the GF probability on the interval (a,b) depends on the behavior of the PDF f(x) on segment (0,a), i.e. $P_{\left(M\right)}\left(a,b\right]$ depends on $f_{1}\left(x\right)$.*

*One can see that the replacement of the function $f_{1}\left(x\right)$ with the function $f_{3}\left(x\right)$ in expression (206) gives that the standard probability P(a,b] will not change, and the nonlocal probability $P_{\left(M\right)}\left(a,b\right]$ can be changed.*

*Other manifestations of nonlocality in the GF PT are described in paper [65].*

**Remark** **20.** *In order to consider a function f(x) as a GF PDF, one should to assume that this function should belong to the set $C_{-1,(K)}^{<1|m>,+}\left(0,\infty \right)$ (see Definition 11). This condition means that the function f(x) can be represented in the form*

(209) $$f\left(x\right)\;=\;I_{\left(K\right)}^{<1|m>,x}\left[u\right]\;\varphi \left(u\right),$$

*where φ(x)≥0 for all x>0. It should be emphasized that the non-negativity of the GF PDF ($f_{\left(K\right)}\left(x\right)\geq 0$ for all x>0) is not enough to obtain the properties I, II, III.*

*If the condition “φ(x)≥0 for all x>0” is not used in the definition of the GF PDF, then one can obtain a nonlocal PT with non-standard properties of the GF CDF and GF probability. For details see paper [65].*

*Let us note that the non-decreasing property of the function $F_{\left(M\right)}\left(x\right)$ (see Property 7) is violated, if the condition $F\left(x\right)\;\in \;C_{-1,(M)}^{<1|m>,+}\left(0,\infty \right)$ without condition $\left(D_{\left(K\right)}^{<1|m>,x}F\right)\left(x\right)\;\in \;C_{-1,(K)}^{<1|m>,+}\left(0,\infty \right)$. In this case, we have only nonlocal (integral) non-decreasing property instead of the local non-decreasing condition. As a result, the non-negativity of the nonlocal probability $P_{\left(M\right)}\left(A\right)$ for all A∈B(Ω), which is defined by the equation*

(210) $$P_{\left(M\right)}\left(x_{1},x_{2}\right]\;:=\;F_{\left(M\right)}\left(x_{2}\right)\;-\;F_{\left(M\right)}\left(x_{1}\right)$$

*is violated for nonlocal CDF $F_{\left(M\right)}\left(x\right)$.*

**Remark** **21.** *Using the fact that probability and functions are dimensionless*

(211) $$\left[P_{\left(M\right)}\left(X\leq x\right)\right]\;=\;\left[F_{\left(M\right)}\left(x\right)\right]\;=\;{\left[x\right]}^{0},$$

*and the equation*

(212) $$F_{\left(M\right)}\left(x\right)\;=\;\left(I_{(M),a+}^{<1|m>}\;f_{\left(K\right)}\right)\left(x\right)\;=\;\int_{a}^{x}M^{<1|m>}\left(x-u\right)\;f_{\left(K\right)}\left(u-a\right)\;du,$$

*one can obtain the dimensions of the GF PDFs of AO $f_{\left(K\right)}\left(x\right)$, the GF CDFs of AO $F_{\left(M\right)}\left(x\right)$. Then, one can obtain the equations*

(213) $$\left[I_{\left(M\right)}^{<1|m>,x}\right]\;=\;\left[I_{x}^{nm}f\left(x\right)\right].$$

(214) $$\left[I_{x}^{n}\right]\;=\;{\left[x\right]}^{n},\;\left[h_{n}\left(x\right)\right]\;=\;{\left[x\right]}^{n-1},$$

*and*

(215) $$\left[M^{<1|m>}\left(x\right)\right]\;=\;{\left[x\right]}^{m\;n-1}.$$

*The dimension of the GF PDFs of AO is given in the form*

(216) $$\left[f_{\left(K\right)}\left(x\right)\right]\;=\;{\left[x\right]}^{-mn}.$$

*For the local case, the standard dimensions are the following*

(217) $$[F\left(x\right)]={\left[x\right]}^{0},\;\left[\int_{0}^{x}f\left(u\right)\;du\right]={\left[x\right]}^{0},\;f\left(x\right)={\left[x\right]}^{-1},$$

*where ${\left[x\right]}^{0}$ means that function is dimensionless.*

## 4. Example of Nonlocal Distribution of AO for Interval (0,∞)

Let us consider the example of nonlocal distribution with the probability distribution function $f_{\left(K\right)}\left(x\right)$ and the kernel $M^{<1|m>}\left(x\right)$ from the set $L_{n}^{<1|m>}$.

One can consider conditions on the parameters, under which the function $f_{\left(K\right)}\left(x\right)$ satisfies the conditions imposed on the nonlocal probability density.

Let $M_{j}\left(x\right)=M_{a|j}\left(x\right)=h_{\alpha_{j}}\left(\lambda \;x\right)$ for all j=1,…,m, where $\alpha_{j}\in \left(0,1\right)$.

Using Proposition 11 of [53], one can consider the kernel $M^{<1|m>}\left(x\right)$ of multi-kernel GFI in the form

(218) $$M^{<1|m>}\left(x\right)\;=\;\lambda^{-(mn-1)}\;h_{\delta +m(n-1)}\left(\lambda \;x\right),$$

where

(219) $$\delta \;=\;\sum_{j=1}^{m}\;\alpha_{j},$$

and $\alpha_{j}\in \left(0,1\right)$ for all j=1,…,m. Equation (218) can be written as

(220) $$M^{<1|m>}\left(x\right)\;=\;\lambda^{-(mn-1)}\;h_{\mu }\left(\lambda \;x\right),$$

where

(221) μ=δ+m(n−1)>0.

The nonlocal probability density for x∈(0,∞) is considered in the form

(222) $$f_{\left(K\right)}\left(x\right)\;=\;\lambda^{mn}\;e_{\alpha ,\beta }\left(\lambda \;x\right)\;=\;\lambda^{mn}\;{\left(\lambda \;x\right)}^{\beta -1}\;E_{\alpha ,\beta }\left[-\;{\left(\lambda \;x\right)}^{\alpha }\right],$$

where α>0, and β∈R. In order for a function (222) to belong to a set $C_{-1}\left(0,\infty \right)$, the condition β>0 must be satisfied.

Let us define the following notation for the Laplace convolution

(223) $$\left(f\left(\lambda \;x\right)\;*\;g\left(\lambda x\right)\right)\;=\;\int_{0}^{x}f\left(\lambda \;\left(x-u\right)\right)\;g\left(\lambda \;u\right)\;du.$$

The nonlocal CDF $F_{\left(M\right)}\left(x\right)$, which is defined as

(224) $$F_{\left(M\right)}\left(x\right)\;=\;I_{\left(M\right)}^{<1|m>,x}\left[u\right]\;f_{\left(K\right)}\left(u\right)\;=\;\left(M^{<1|m>}\;*\;f_{\left(K\right)}\right)\left(x\right)\;=\;\int_{0}^{x}M^{<1|m>}\left(x\;-\;u\right)\;f_{\left(K\right)}\left(u\right)\;du,$$

is described by the expression

(225) $$F_{\left(M\right)}\left(x\right)\;=\;\left(\lambda^{mn}\;h_{\mu }\left(\lambda \;x\right)\;*\;\lambda^{-(mn-1)}\;e_{\alpha ,\beta }\left(\lambda \;x\right)\right)\;=\;\lambda \;\left(h_{\mu }\left(\lambda \;x\right)\;*\;e_{\alpha ,\beta }\left(\lambda \;x\right)\right).$$

Then, using Equation 4.4.5 of [85], p. 61, in the form

(226) $$\left(h_{\mu }\left(\lambda \;x\right)\;*\;e_{\alpha ,\beta }\left(\lambda \;x\right)\right)\;=\;\lambda^{-1}\;e_{\alpha ,\beta +\mu }\left(\lambda \;x\right),$$

where μ>0, β>0, Equation (225) takes the form

(227) $$F_{\left(M\right)}\left(x\right)\;=\;\lambda \;\lambda^{-1}\;e_{\alpha ,\beta +\mu }\left(\lambda \;x\right)\;=\;e_{\alpha ,\beta +\mu }\left(\lambda \;x\right),$$

where it is assumed that the parameters satisfy the conditions

(228) μ>0,α>0,β>0.

Let us find the restrictions on the parameters α, β, μ under which the conditions

(229) $$\lim_{x\to 0+}F_{\left(M\right)}\left(x\right)\;=\;0,$$

(230) $$\lim_{x\to \infty }F_{\left(M\right)}\left(x\right)\;=\;1$$

are satisfied for function (227).

Using the definition of the two-parameter Mittag–Leffler function by Equation (4).1.1 of [85], p. 56, in the form

(231) $$E_{\alpha ,\beta }\left[z\right]\;=\;\sum_{k=0}^{\infty }\frac{z^{k}}{\Gamma (\alpha \;k\;+\;\beta )}\;=\;\frac{1}{\Gamma (\beta )}\;+\;\sum_{k=1}^{\infty }\frac{z^{k}}{\Gamma (\alpha \;k\;+\;\beta )},$$

where α>0 and β∈R, one can see that

(232) $$\lim_{x\to 0+}\;F_{\left(M\right)}\left(x\right)\;=\;\lim_{x\to 0+}\;{\left(\lambda \;x\right)}^{\beta -1+\mu }\;\frac{1}{\Gamma (\beta )}\;+\;\lim_{x\to 0+}\;\sum_{k=1}^{\infty }\frac{{\left(-1\right)}^{k}\;{\left(\lambda \;x\right)}^{k\alpha +\beta -1+\mu }}{\Gamma (\alpha \;k\;+\;\beta )}.$$

Therefore, property (229) is satisfied, if the inequality

(233) β−1+μ>0

holds.

To prove property (230), one can use Theorem 4.3 of [85], p. 64, which gives the asymptotic equation

(234) $$E_{\alpha ,\beta }\left[-\;z\right]\;=\;-\;\sum_{k=1}^{m}\frac{1}{\Gamma (\beta -k\;\alpha )}\;\frac{1}{{\left(-z\right)}^{k}}\;+{\;O(|z|}^{-m-1})\;(|z|\;\to \;\infty ).$$

For example, Equation (234) holds for the case 0<α<2 [4], p. 43. Using (234), function (227) satisfies the following asymptotic equation

(235) $$\begin{array}{c} F_{\left(M\right)}\left(x\right)\;=\;{\left(\lambda \;x\right)}^{\beta -1+\mu }\;E_{\alpha ,\beta +\mu }\left[-\;{\left(\lambda \;x\right)}^{\alpha }\right]\;=\;\\ \frac{1}{\Gamma (\beta +\mu \;-\;\alpha )}\;\frac{{\left(\lambda \;x\right)}^{\beta -1+\mu }}{{\left(\lambda \;x\right)}^{\alpha }}\;+\;O\left(x^{\beta -1+\mu -2\alpha }\right)\;=\;\\ \frac{1}{\Gamma (\beta +\mu \;-\;\alpha )}\;{\left(\lambda \;x\right)}^{\beta -1+\mu -\alpha }\;+\;O\left(x^{\beta -1+\mu -2\alpha }\right) \end{array}$$

for x→∞.

As a result, property (230) holds, if the following equality is satisfied

(236) β−1+μ−α=0,

where the condition 0<α<2 is assumed.

Using condition (236) and Γ(1), Equation (235) takes the form

(237) $$F_{\left(M\right)}\left(x\right)\;=\;1\;+\;O\left(x^{\beta -1+\mu -2\alpha }\right)\;=\;1\;+\;O\left(x^{-\alpha }\right)$$

for x→∞, since β−1+μ−2α=−α<0. Therefore, property (230) is satisfied, and $F_{\left(M\right)}\left(x\right)\;\to \;1$ at x→∞.

For case (236), inequality (233), which is used for $F_{\left(M\right)}\left(0+\right)=0$, is satisfied, since

(238) β−1+μ=α>0.

Condition allows to consider a wider range of parameters of the nonlocal probability distributions compared to article [65].

As a result, the following proposition for nonlocal distribution on (0,∞) is proved.

**Proposition** **3.** *Let $M_{j}\left(x\right)=M_{a|j}\left(x\right)=h_{\alpha_{j}}\left(\lambda \;x\right)$ for all j=1,…,m, where $\alpha_{j}\in \left(0,1\right)$.*

*Using Proposition 11 of [53], one can consider the kernel $M^{<1|m>}\left(x\right)$ of multi-kernel GFI in the form*

(239) $$M^{<1|m>}\left(x\right)\;=\;\lambda^{-(mn-1)}\;h_{\delta +m(n-1)}\left(\lambda \;x\right),$$

*where*

(240) $$\delta \;=\;\sum_{j=1}^{m}\;\alpha_{j},$$

*and $\alpha_{j}\in \left(0,1\right)$ for all j=1,…,m.*

*Then, the following function $f_{\left(K\right)}\left(x\right)$ with x∈(0,∞) is the nonlocal PDF of AO*

(241) $$f_{\left(K\right)}\left(x\right)\;=\;\lambda \;{\left(\lambda \;x\right)}^{\beta -1}\;E_{\alpha ,\beta }\left[-\;{\left(\lambda \;x\right)}^{\alpha }\right],$$

*if*

(242) δ+m(n−1)+β−α−1=0,

*where α∈(0,2), and β>0.*

*The nonlocal CDF for x∈(0,∞) has the form*

(243) $$F_{\left(M\right)}\left(x\right)\;=\;e_{\alpha ,\beta +\mu }\left(\lambda \;x\right)\;=\;{\left(\lambda \;x\right)}^{\beta \delta \;+\;m(n-1)-1}\;E_{\alpha ,\beta +\delta \;+\;m(n-1)}\left[-\;{\left(\lambda \;x\right)}^{\alpha }\right],$$

*where μ=δ+m(n−1)>0*

## 5. Examples of Nonlocal Distributions on Finite Interval [a,b]

### 5.1. GFIs for x>a>−∞

To derive nonlocal (GF) distributions of AO on the finite intervals [a,b], where −∞<a<b<∞, and nonlocal CDFs $F_{\left(M\right)}\left(x\right)$ of AO, the following equations can be used.

(244) $$\int_{a}^{x}\;du\;h_{\alpha }\left(\lambda \;\left(x-u\right)\right)\;h_{\beta }\left(\lambda \;\left(u-a\right)\right)\;=\;\lambda^{-1}\;h_{\alpha +\beta }\left(\lambda \;\left(x-a\right)\right).$$

(245) $$\int_{a}^{x}\;du\;h_{\alpha }\left(\lambda \;\left(x-u\right)\right)\;h_{\beta ,\lambda }\left(\lambda \;\left(x-a\right)\right)\;=\;\lambda^{-1}\;\phi_{\beta ,\alpha +\beta }\left(\lambda \;\left(x-a\right)\right).$$

(246) $$\int_{a}^{x}\;du\;h_{\mu }\left(\lambda \;\left(x-u\right)\right)\;e_{\alpha ,\beta }\left(\lambda \;\left(x-a\right)\right)\;=\;\lambda^{-1}\;e_{\alpha ,\beta +\mu }\left(\lambda \;\left(x-a\right)\right).$$

(247) $$\int_{a}^{x}\;du\;h_{\mu }\left(\lambda \;\left(x-u\right)\right)\;\omega_{\alpha }\left(\lambda \;\left(x-a\right)\right)\;=\;\lambda^{-1}\;\omega_{\alpha +\mu }\left(\lambda \;\left(x-a\right)\right).$$

(248) $$\int_{a}^{x}\;du\;h_{\mu }\left(\lambda \;\left(x-u\right)\right)\;\phi_{\alpha ,\beta }\left(\lambda \;\left(x-a\right)\right)\;=\;\lambda^{-1}\;\phi_{\alpha ,\beta +\mu }\left(\lambda \;\left(x-a\right)\right).$$

In Equations (244)–(248), one can consider

(249) −∞<a<x≤b≤∞,

and the positive parameters α>0, β>0, μ>0, λ>0.

Note that in these equations, both the first and second integrands can be considered as nonlocal (GF) PDFs on the interval [a,b] up to numerical factors, if the appropriate parameter ranges are used.

### 5.2. Examples of Nonlocal Distributions for Power-Law Nonlocality

Using Propositions 11–16 of [53], pp. 27–30, and Equations (244)–(248), one can obtain the following examples of nonlocal (GF) distributions.

**Example** **1.** *Let $\left(M_{j},K_{j}\right)$ belong to the Luchko set $L_{1}$ such that $M_{j}\left(x\right)=h_{\alpha_{j}}\left(\lambda \;x\right)$ for all j=1,…,m, where $\alpha_{j}\in \left(0,1\right)$.*

*Using Proposition 11 of [53] and Equation (244), one can obtain the following nonlocal (GF) distribution on the interval [a,b], where −∞<a<b<∞.*

*For the kernel of the multi-kernel GFI*

(250) $$M_{a}^{<1|m>}\left(x\right)\;=\;\lambda^{-mn+1}\;h_{\alpha +m(n-1)}\left(\lambda \;x\right),$$

*and the nonlocal PDF*

(251) $$f_{\left(K\right)}\left(x\right)\;=\;\lambda^{mn}\;\frac{h_{\beta }\left(\lambda \;\left(x\;-\;a\right)\right)}{h_{\alpha +\beta +m(n-1)}\left(\lambda \;\left(b-a\right)\right)},$$

*on the interval [a,b], the nonlocal CDF has the form*

(252) $$F_{\left(M\right)}\left(x\right)\;=\;\frac{h_{\alpha +\beta +m(n-1)}\left(\lambda \;\left(x-a\right)\right)}{h_{\alpha +\beta +m(n-1)}\left(\lambda \;\left(b-a\right)\right)},$$

*where λ>0, β>0, and*

(253) $$\alpha \;=\;\sum_{j=1}^{m}\;\alpha_{j},\;\alpha_{j}\in \left(0,1\right)\;for\;all\;j\;=\;1,\;\ldots ,\;m.$$

**Example** **2.** *Let $\left(M_{j},K_{j}\right)$ belong to the Luchko set $L_{1}$ such that $M_{j}\left(x\right)=h_{\alpha_{j}}\left(\lambda \;x\right)$ for all j=1,…,m, where $\alpha_{j}\in \left(0,1\right)$.*

*Using Proposition 11 of [53] and Equation (245), one can obtain the following nonlocal (GF) distribution on the interval [a,b], where −∞<a<b<∞.*

*For the kernel of the multi-kernel GFI*

(254) $$M_{a}^{<1|m>}\left(x\right)\;=\;\lambda^{-mn+1}\;h_{\alpha +m(n-1)}\left(\lambda \;x\right),$$

*and the nonlocal PDF*

(255) $$f_{\left(K\right)}\left(x\right)\;=\;\lambda^{mn}\;\frac{h_{\beta ,\lambda }\left(\lambda \;\left(x\;-\;a\right)\right)}{\phi_{\beta ,\alpha +\beta +m(n-1)}\left(\lambda \;\left(b-a\right)\right)},$$

*on the interval [a,b], the nonlocal CDF has the form*

(256) $$F_{\left(M\right)}\left(x\right)\;=\;\frac{\phi_{\beta ,\alpha +\beta +m(n-1)}\left(\lambda \;\left(x-a\right)\right)}{\phi_{\beta ,\alpha +\beta +m(n-1)}\left(\lambda \;\left(b-a\right)\right)},$$

*where λ>0, β>0, and*

(257) $$\alpha \;=\;\sum_{j=1}^{m}\;\alpha_{j},\;\alpha_{j}\in \left(0,1\right)\;for\;all\;j\;=\;1,\;\ldots ,\;m.$$

**Example** **3.** *Let $\left(M_{j},K_{j}\right)$ belong to the Luchko set $L_{1}$ such that $M_{j}\left(x\right)=h_{\alpha_{j}}\left(\lambda \;x\right)$ for all j=1,…,m, where $\alpha_{j}\in \left(0,1\right)$.*

*Using Proposition 11 of [53] and Equation (246), one can obtain the following nonlocal (GF) distribution on the interval [a,b], where −∞<a<b<∞.*

*For the kernel of the multi-kernel GFI*

(258) $$M_{a}^{<1|m>}\left(x\right)\;=\;\lambda^{-mn+1}\;h_{\alpha +m(n-1)}\left(\lambda \;x\right),$$

*and the nonlocal PDF*

(259) $$f_{\left(K\right)}\left(x\right)\;=\;\lambda^{mn}\;\frac{e_{\delta ,\beta }\left(\lambda \;\left(x\;-\;a\right)\right)}{e_{\delta ,\beta +\alpha +m(n-1)}\left(\lambda \;\left(b-a\right)\right)}$$

*on the interval [a,b], the nonlocal CDF has the form*

(260) $$F_{\left(M\right)}\left(x\right)\;=\;\frac{e_{\delta ,\beta +\alpha +m(n-1)}\left(\lambda \;\left(x-a\right)\right)}{e_{\delta ,\beta +\alpha +m(n-1)}\left(\lambda \;\left(b-a\right)\right)},$$

*where λ>0, β>0, δ>0, and*

(261) $$\alpha \;=\;\sum_{j=1}^{m}\;\alpha_{j},\;\alpha_{j}\in \left(0,1\right)\;for\;all\;j\;=\;1,\;\ldots ,\;m.$$

*As a special case, one can consider $e_{1,1}\left(x\right)=\exp\left(-x\right)$. It should be noted that the function $e_{\delta ,\beta }\left(\lambda \;x\right)$ belongs to the set $L_{1}$ is 0<δ≤β<1. Therefore $e_{1,1}\left(x\right)=\exp\left(-x\right)$ cannot belong to the set $L_{1}$. In this case (δ=β=1) and kernel (258), the nonlocal PDF (259) takes the form*

(262) $$f_{\left(K\right)}\left(x\right)\;=\;\lambda^{mn}\;\frac{\exp(-\;\lambda \;(x\;-\;a))}{e_{1,1+\alpha +m(n-1)}\left(\lambda \;\left(b-a\right)\right)}$$

*on the interval [a,b], the nonlocal CDF takes the form*

(263) $$F_{\left(M\right)}\left(x\right)\;=\;\frac{e_{1,1+\alpha +m(n-1)}\left(\lambda \;\left(x-a\right)\right)}{e_{1,1+\alpha +m(n-1)}\left(\lambda \;\left(b-a\right)\right)},$$

**Example** **4.** *Let $\left(M_{j},K_{j}\right)$ belong to the Luchko set $L_{1}$ such that $M_{j}\left(x\right)=h_{\alpha_{j}}\left(\lambda \;x\right)$ for all j=1,…,m, where $\alpha_{j}\in \left(0,1\right)$.*

*Using Proposition 11 of [53] and Equation (247), one can obtain the following nonlocal (GF) distribution on the interval [a,b], where −∞<a<b<∞.*

*For the kernel of the multi-kernel GFI*

(264) $$M_{a}^{<1|m>}\left(x\right)\;=\;\lambda^{-mn+1}\;h_{\alpha +m(n-1)}\left(\lambda \;x\right),$$

*and the nonlocal PDF*

(265) $$f_{\left(K\right)}\left(x\right)\;=\;\lambda^{mn}\;\frac{\omega_{\beta }\left(\lambda \;\left(x\;-\;a\right)\right)}{\omega_{\beta +\alpha +m(n-1)}\left(\lambda \;\left(b-a\right)\right)}$$

*on the interval [a,b], the nonlocal CDF has the form*

(266) $$F_{\left(M\right)}\left(x\right)\;=\;\frac{\omega_{\beta +\alpha +m(n-1)}\left(\lambda \;\left(x-a\right)\right)}{\omega_{\beta +\alpha +m(n-1)}\left(\lambda \;\left(b-a\right)\right)},$$

*where λ>0, β>0, and*

(267) $$\alpha \;=\;\sum_{j=1}^{m}\;\alpha_{j},\;\alpha_{j}\in \left(0,1\right)\;for\;all\;j\;=\;1,\;\ldots ,\;m.$$

**Example** **5.** *Let $\left(M_{j},K_{j}\right)$ belong to the Luchko set $L_{1}$ such that $M_{j}\left(x\right)=h_{\alpha_{j}}\left(\lambda \;x\right)$ for all j=1,…,m, where $\alpha_{j}\in \left(0,1\right)$.*

*Using Proposition 11 of [53] and Equation (248), one can obtain the following nonlocal (GF) distribution on the interval [a,b], where −∞<a<b<∞.*

*For the kernel of the multi-kernel GFI*

(268) $$M_{a}^{<1|m>}\left(x\right)\;=\;\lambda^{-mn+1}\;h_{\alpha +m(n-1)}\left(\lambda \;x\right),$$

*and the nonlocal PDF*

(269) $$f_{\left(K\right)}\left(x\right)\;=\;\lambda^{mn}\;\frac{\phi_{\delta ,\beta }\left(\lambda \;\left(x\;-\;a\right)\right)}{\phi_{\delta ,\beta +\alpha +m(n-1)}\left(\lambda \;\left(b-a\right)\right)}$$

*on the interval [a,b], the nonlocal CDF has the form*

(270) $$F_{\left(M\right)}\left(x\right)\;=\;\frac{\phi_{\delta ,\beta +\alpha +m(n-1)}\left(\lambda \;\left(x-a\right)\right)}{\phi_{\delta ,\beta +\alpha +m(n-1)}\left(\lambda \;\left(b-a\right)\right)},$$

*where λ>0, β>0, δ>0 and*

(271) $$\alpha \;=\;\sum_{j=1}^{m}\;\alpha_{j},\;\alpha_{j}\in \left(0,1\right)\;\mathrm{for}\;\mathrm{all}\;j\;=\;1,\;\ldots ,\;m.$$

**Example** **6.** *Let $\left(M_{j},K_{j}\right)$ belong to the Luchko set $L_{1}$ such that $M_{j}\left(x\right)=h_{\alpha_{j}}\left(\lambda \;x\right)$ for all j=1,…,m, where $\alpha_{j}\in \left(0,1\right)$. Using Proposition 11 of [53] and Equation (22) of Table 9.1 in [1], p. 173, in the form*

(272) $$\int_{a}^{x}h_{\alpha }\left(x-u\right)\;\psi_{\mu ,\nu ,\beta }\left(\lambda \;\left(x-a\right)\right)\;=\;\psi_{\mu ,\nu ,\beta +\alpha }\left(\lambda \;\left(x-a\right)\right),$$

*where*

(273) $$\psi_{\mu ,\nu ,\beta }\left(\lambda \;x\right)\;:=\;\frac{{\left(\lambda \;x\right)}^{\beta -1}}{\Gamma (\beta )}{\;}_{2}F_{1}\left(\mu ,\nu ;\beta ;-\;\lambda \;x\right),$$

*one can obtain the following nonlocal (GF) distribution on the interval [a,b], where −∞<a<b<∞.*

*For the kernel of the multi-kernel GFI*

(274) $$M_{a}^{<1|m>}\left(x\right)\;=\;\lambda^{-mn+1}\;h_{\alpha +m(n-1)}\left(\lambda \;x\right),$$

*and the nonlocal PDF*

(275) $$f_{\left(K\right)}\left(x\right)\;=\;\lambda^{mn}\;\frac{\psi_{\mu ,\nu ,\beta }\left(\lambda \;\left(x\;-\;a\right)\right)}{\psi_{\mu ,\nu ,\beta +\alpha }\left(\lambda \;\left(b-a\right)\right)}$$

*on the interval [a,b], the nonlocal CDF has the form*

(276) $$F_{\left(M\right)}\left(x\right)\;=\;\frac{\psi_{\mu ,\nu ,\beta +\alpha }\left(\lambda \;\left(x-a\right)\right)}{\psi_{\mu ,\nu ,\beta +\alpha }\left(\lambda \;\left(b-a\right)\right)},$$

*where λ>0, β>0, μ>0, ν>0, and*

(277) $$\alpha \;=\;\sum_{j=1}^{m}\;\alpha_{j},\;\alpha_{j}\in \left(0,1\right)\;for\;all\;j\;=\;1,\;\ldots ,\;m.$$

### 5.3. Examples of Nonlocal Distributions for Other Types of Nonlocality

In this subsection, the nonlocal (GF) distributions of AO are considered for non-localities of non-power-law types.

**Example** **7.** *Let $\left(M_{j},K_{j}\right)$ belong to the Luchko set $L_{1}$ such that $M_{j}\left(x\right)=h_{\alpha_{j},\lambda }\left(\lambda \;x\right)$ for all j=1,…,m, where $\alpha_{j}\in \left(0,1\right)$.*

*Using Proposition 12 of [53] and Equation (248), one can obtain the following nonlocal (GF) distribution on the interval [a,b], where −∞<a<b<∞.*

*For the kernel of the multi-kernel GFI*

(278) $$M_{b}^{<1|m>}\left(x\right)\;=\;\lambda^{-mn+1}\;\phi_{\alpha ,\alpha +m(n-1)}\left(\lambda \;x\right),$$

*and the nonlocal PDF*

(279) $$f_{\left(K\right)}\left(x\right)\;=\;\lambda^{mn}\;\frac{h_{\beta }\left(\lambda \;\left(x\;-\;a\right)\right)}{\phi_{\alpha ,\alpha +\beta +m(n-1)}\left(\lambda \;\left(b-a\right)\right)},$$

*on the interval [a,b], the nonlocal CDF has the form*

(280) $$F_{\left(M\right)}\left(x\right)\;=\;\frac{\phi_{\alpha ,\alpha +\beta +m(n-1)}\left(\lambda \;\left(x-a\right)\right)}{\phi_{\alpha ,\alpha +\beta +m(n-1)}\left(\lambda \;\left(b-a\right)\right)},$$

*where λ>0, and*

(281) $$\alpha \;=\;\sum_{j=1}^{m}\;\alpha_{j},\;\alpha_{j}\in \left(0,1\right)\;\mathrm{for}\;\mathrm{all}\;j\;=\;1,\;\ldots ,\;m.$$

**Example** **8.** *Let $\left(M_{j},K_{j}\right)$ belong to the Luchko set $L_{1}$ such that $M_{j}\left(x\right)=h_{\alpha_{j}}\left(\lambda \;x\right)$ for all j=1,…,(m−1), where $\alpha_{j}\in \left(0,1\right)$, and let $M_{m}\left(x\right)=e_{\alpha ,\beta }\left(\lambda \;x\right)$ for j=m, where 0<α≤β<1.*

*Using Proposition 13 of [53] and Equation (246), one can obtain the following nonlocal (GF) distribution on the interval [a,b], where −∞<a<b<∞.*

*For the kernel of the multi-kernel GFI*

(282) $$M_{c}^{<1|m>}\left(x\right)\;=\;\lambda^{-(mn-1)}\;e_{\alpha ,\beta +\mu +m(n-1)}\left(\lambda \;x\right)),$$

*and the nonlocal PDF*

(283) $$f_{\left(K\right)}\left(x\right)\;=\;\lambda^{mn}\;\frac{h_{\delta }\left(\lambda \;\left(x\;-\;a\right)\right)}{e_{\alpha ,\beta +\delta +m(n-1)}\left(\lambda \;\left(b-a\right)\right)}$$

*on the interval [a,b], the nonlocal CDF has the form*

(284) $$F_{\left(M\right)}\left(x\right)\;=\;\frac{e_{\alpha ,\beta +\delta +m(n-1)}\left(\lambda \;\left(x-a\right)\right)}{e_{\alpha ,\beta +\delta +m(n-1)}\left(\lambda \;\left(b-a\right)\right)},$$

*where λ>0, 0<α≤β<1, δ>0, and*

(285) $$\mu \;=\;\sum_{j=1}^{m-1}\;\alpha_{j},\;\alpha_{j}\in \left(0,1\right)\;for\;all\;j\;=\;1,\;\ldots ,\;(m-1).$$

**Example** **9.** *Let $\left(M_{j},K_{j}\right)$ belong to the Luchko set $L_{1}$ such that $M_{j}\left(x\right)=h_{\alpha_{j}}\left(\lambda \;x\right)$ for all j=1,…,(m−1), where $\alpha_{j}\in \left(0,1\right)$, and let $M_{m}\left(x\right)=\omega_{\alpha }\left(\lambda \;x\right)$ for j=m, where 0<α<1.*

*Using Proposition 14 of [53] and Equation (247), one can obtain the following nonlocal (GF) distribution on the interval [a,b], where −∞<a<b<∞.*

*For the kernel of the multi-kernel GFI*

(286) $$M_{d}^{<1|m>}\left(x\right)\;=\;\lambda^{-(mn-1)}\;\omega_{\alpha +\mu +m(n-1)}\left(\lambda \;x\right)),$$

*and the nonlocal PDF*

(287) $$f_{\left(K\right)}\left(x\right)\;=\;\lambda^{mn}\;\frac{h_{\delta }\left(\lambda \;\left(x\;-\;a\right)\right)}{\omega_{\alpha +\delta +m(n-1)}\left(\lambda \;\left(b-a\right)\right)}$$

*on the interval [a,b], the nonlocal CDF has the form*

(288) $$F_{\left(M\right)}\left(x\right)\;=\;\frac{\omega_{\alpha +\delta +m(n-1)}\left(\lambda \;\left(x-a\right)\right)}{\omega_{\alpha +\delta +m(n-1)}\left(\lambda \;\left(b-a\right)\right)},$$

*where λ>0, 0<α<1, δ>0, and*

(289) $$\mu \;=\;\sum_{j=1}^{m-1}\;\alpha_{j},\;\alpha_{j}\in \left(0,1\right)\;\mathrm{for}\;\mathrm{all}\;j\;=\;1,\;\ldots ,\;(m-1).$$

**Example** **10.** *Let $\left(M_{j},K_{j}\right)$ belong to the Luchko set $L_{1}$ such that $M_{j}\left(x\right)=h_{\alpha_{j}}\left(\lambda \;x\right)$ for all j=1,…,k, and let $M_{j}\left(x\right)=h_{\alpha_{j},\lambda }\left(\lambda \;x\right)$ for all j=k+1,…,m), where $\beta_{j}\;in\;\;\left(0,1\right)$ for all j=1,…,m.*

*Using Proposition 15 of [53] and Equation (248), one can obtain the following nonlocal (GF) distribution on the interval [a,b], where −∞<a<b<∞.*

*For the kernel of the multi-kernel GFI*

(290) $$M_{f}^{<1|m>}\left(x\right)\;=\;\lambda^{-(mn-1)}\;\phi_{\nu ,m(n-1)+\eta +\nu }\left(\lambda \;x\right),$$

*and the nonlocal PDF*

(291) $$f_{\left(K\right)}\left(x\right)\;=\;\lambda^{mn}\;\frac{h_{\delta }\left(\lambda \;\left(x\;-\;a\right)\right)}{\phi_{\nu ,m(n-1)+\eta +\delta +\nu }\left(\lambda \;\left(b-a\right)\right)}$$

*on the interval [a,b], the nonlocal CDF has the form*

(292) $$F_{\left(M\right)}\left(x\right)\;=\;\frac{\phi_{\nu ,m(n-1)+\eta +\delta +\nu }\left(\lambda \;\left(x-a\right)\right)}{\phi_{\nu ,m(n-1)+\eta +\delta +\nu }\left(\lambda \;\left(b-a\right)\right)},$$

*where λ>0, 0<α≤β<1, δ>0, and*

(293) $$\eta \;=\;\sum_{j=1}^{k}\;\alpha_{j},\;\nu \;=\;\sum_{j=k+1}^{m}\;\alpha_{j},\;\alpha_{j}\in \left(0,1\right)\;\mathrm{for}\;\mathrm{all}\;j\;=\;1,\;\ldots ,\;m.$$

**Example** **11.** *Let $\left(M_{j},K_{j}\right)$ belong to the Luchko set $L_{1}$ such that $M_{j}\left(x\right)=h_{\alpha_{j}}\left(\lambda \;x\right)$ for all j=1,…,(m−1), where $\alpha_{j}\in \left(0,1\right)$, and let $M_{m}\left(x\right)=\phi_{\alpha ,\beta }\left(\lambda \;x\right)$ for j=m, where 0<α≤β<1.*

*Using Proposition 16 of [53] and Equation (248), one can obtain the following nonlocal (GF) distribution on the interval [a,b], where −∞<a<b<∞.*

*For the kernel of the multi-kernel GFI*

(294) $$M_{g}^{<1|m>}\left(x\right)\;=\;\lambda^{-(mn-1)}\;\phi_{\alpha ,\beta +\mu +m(n-1)}\left(\lambda \;x\right),$$

*and the nonlocal PDF*

(295) $$f_{\left(K\right)}\left(x\right)\;=\;\lambda^{mn}\;\frac{h_{\delta }\left(\lambda \;\left(x\;-\;a\right)\right)}{\phi_{\alpha ,\beta +\delta +m(n-1)}\left(\lambda \;\left(b-a\right)\right)}$$

*on the interval [a,b], the nonlocal CDF has the form*

(296) $$F_{\left(M\right)}\left(x\right)\;=\;\frac{\phi_{\alpha ,\beta +\delta +m(n-1)}\left(\lambda \;\left(x-a\right)\right)}{\phi_{\alpha ,\beta +\delta +m(n-1)}\left(\lambda \;\left(b-a\right)\right)},$$

*where λ>0, 0<α≤β<1, δ>0, and*

(297) $$\mu \;=\;\sum_{j=1}^{m-1}\;\alpha_{j},\;\alpha_{j}\in \left(0,1\right)\;for\;all\;j\;=\;1,\;\ldots ,\;(m-1).$$

Note that nonlocal probability distributions are not restricted by the examples that are described in this section.

## 6. Conclusions

Let us briefly list the main results obtained in this article.

(1) In this paper, nonlocal PT of AO is proposed as a generalizations of the nonlocal PT of “first-order”, which is suggested in [65], and fractional PT of AO, which is proposed for power-law type nonlocality in paper [81]. In the proposed extension of standard PT, the multi-kernel GFC of AO, which is suggested in [53], are used.

(2) Basic concepts of nonlocal PT of AO are proposed by using the multi-kernel GFC of AO on intervals (0,∞) and (a,b) with −∞<a<b<∞. As a result, operator kernels, which describe nonlocalities, belong to a wider set of functions than kernels in the single-kernel Luchko GFC.

(3) The nonlocal (GF) PDFs, nonlocal (GF) CDFs and nonlocal (GF) probability are defined and its properties are described. In this proposed approach to PT, the nonlocal (GF) CDFs and nonlocal (GF)PDFs are defined as two pairs of functions (GFD, PDF) and operator kernels (kernels of GFI and GFD). The characteristic properties of these pairs aree used to construct nonlocal and GF probability and probability spaces, where the functions that define nonlocality in space are taken into account as an additional structure.

(4) The construction of the nonlocal PT of AO is based on an application and generalization of the well-known theorem of PT, according to which the existence of function satisfies the characteristic properties of CDF leads to the existence of unique probability space (see [88], p. 35, and [87], p. 185). The correspondence between probability measures and CDFs is used to construct GF probability and GF probability spaces by specifying the corresponding nonlocal (GF) CDFs.

(5) Examples of nonlocal probability distributions of AO on intervals (0,∞) and (a,b) with −∞<a<b<∞ are described.

It should be noted that the proposed nonlocal PT cannot be reduced to a standard PT, just as that fractional calculus and GFC cannot be reduced to the standard calculus of integrals and derivatives of arbitrary integer orders.

As a future development of the nonlocal and GF PT, the following directions may be of interest.

(a) It is important to expand the proposed nonlocal an GF PT to high dimension on $R^{n}$, and bounded domains in $mathbbR^{n}$ with integer n>1 by using [52,82]. Note that the general fractional calculus of many variables, which is partially described in [52], can be used for detailed study of GF distributions in multidimensional spaces. The definitions of *n*-dimensional GF operators should be formulated in orthogonal curvilinear coordinates by using the Lame coefficients [52].

(b) It is important to write mathematically accurately the description of nonlocal probabilities for piecewise continuous functions that describes nonlocal and GF CDF and PDF. One can assume that the piecewise continuous case can be described by using some of the tools used in paper [52].

(c) It is important to have a nonlocal PT on the entire real axis, and not just on the positive semiaxis. Note that such formulations clearly go beyond the function spaces used in the Luchko form of the GFC.

(d) It is interesting to consider new types of nonlocalities, for which mathematically correct nonlocal (GF) PT can be formulated. For example, one can consider GFC based on the nonlocalities described by the Fourier convolution [67] and the Mellin convolution [66] in addition to the Luchko GFC, which is based on the Laplace convolution.

(e) One can important to formulate discrete analogues of nonlocal and general fractional PT that mathematically correct describe nonlocal discrete distributions. Unfortunately, a discrete analogue of the Luchko GFC has not been created at the present time. Such formulations of GFC should include the well-known Riesz-type fractional operators and Hadamard-type fractional operators [4].

An application of the multi-kernel GFC allows us to use a wider class of operator kernels in GFIs and GFDs. As a result, one can consider a wider class of nonlocalities in models of nonlocal statistical mechanics (see paper [80] and references therein), in physical kinetics and nonlocal quantum physics, statistical optics. Nonlocality in physical processes can manifest itself in the form of non-standard frequency and spatial dispersions. The proposed extension of the probability theory may be useful in economics and technical sciences.

## Data Availability

Not applicable.
